# The use of nonnormalized surface EMG and feature inputs for LSTM-based powered ankle prosthesis control algorithm development

**DOI:** 10.3389/fnins.2023.1158280

**Published:** 2023-07-03

**Authors:** Ahmet Doğukan Keleş, Ramazan Tarık Türksoy, Can A. Yucesoy

**Affiliations:** ^1^Institute of Biomedical Engineering, Boğaziçi University, Istanbul, Türkiye; ^2^Institute for Modelling and Simulation of Biomechanical Systems, University of Stuttgart, Stuttgart, Germany; ^3^Huawei Turkey R&D Center, Istanbul, Türkiye

**Keywords:** powered ankle prosthesis, surface electromyogram (sEMG), long short-term memory neural network, feature extraction, lower limb amputation

## Abstract

Advancements in instrumentation support improved powered ankle prostheses hardware development. However, control algorithms have limitations regarding number and type of sensors utilized and achieving autonomous adaptation, which is key to a natural ambulation. Surface electromyogram (sEMG) sensors are promising. With a minimized number of sEMG inputs an *economic* control algorithm can be developed, whereas limiting the use of lower leg muscles will provide a *practical* algorithm for both ankle disarticulation and transtibial amputation. To determine appropriate sensor combinations, a systematic assessment of the predictive success of variations of multiple sEMG inputs in estimating ankle position and moment has to conducted. More importantly, tackling the use of nonnormalized sEMG data in such algorithm development to overcome processing complexities in real-time is essential, but lacking. We used healthy population level walking data to (1) develop sagittal ankle position and moment predicting algorithms using nonnormalized sEMG, and (2) rank all muscle combinations based on success to determine economic and practical algorithms. Eight lower extremity muscles were studied as sEMG inputs to a long-short-term memory (LSTM) neural network architecture: tibialis anterior (TA), soleus (SO), medial gastrocnemius (MG), peroneus longus (PL), rectus femoris (RF), vastus medialis (VM), biceps femoris (BF) and gluteus maximus (GMax). Five features extracted from nonnormalized sEMG amplitudes were used: integrated EMG (IEMG), mean absolute value (MAV), Willison amplitude (WAMP), root mean square (RMS) and waveform length (WL). Muscle and feature combination variations were ranked using Pearson’s correlation coefficient (r > 0.90 indicates successful correlations), the root-mean-square error and one-dimensional statistical parametric mapping between the original data and LSTM response. The results showed that IEMG+WL yields the best feature combination performance. The best performing variation was MG + RF + VM (*r*_position_ = 0.9099 and *r*_moment_ = 0.9707) whereas, PL (*r*_position_ = 0.9001, *r*_moment_ = 0.9703) and GMax+VM (*r*_position_ = 0.9010, *r*_moment_ = 0.9718) were distinguished as the economic and practical variations, respectively. The study established for the first time the use of nonnormalized sEMG in control algorithm development for level walking.

## 1. Introduction

Lower limb amputations include transtibial amputation, i.e., a surgical procedure to fully remove the lower limb below the knee, and ankle disarticulation, i.e., surgical removal of the foot at the ankle joint. The incidence rates of these interventions are high, reaching, e.g., over 11,500 cases alone in the UK each year (Isaacs-Itua and Sedki, 2018) and from 2003 to 2013, about half of those were transtibial amputations (Ahmad et al., 2016). Ascribed to a simple, low economic cost and robust design, energy storing and returning prostheses with elastic and damping characteristics (Brackx et al., 2013) dominate the commercial lower limb prostheses market (Gehlhar et al., 2020). However, because of lack of mechanical power generation and autonomous adaptation they can only provide lower than normal gait speeds with high metabolic energy costs that leads to early fatigue (Vucina et al., 2005; Brackx et al., 2013; Xu et al., 2021). Central to these limitations is the absence of a sensory feedback to characterize user dependent needs (Tucker et al., 2015).

Current studies on powered prostheses, which devices overcome the indicated shortcomings and assist users’ gait at walking speeds close to normal (Nasr et al., 2021) do utilize several sensors including inertial measurement units (Culver et al., 2018; Shultz and Goldfarb, 2018), pressure sensors (Attal et al., 2018; Liu et al., 2019), force sensors (Sup et al., 2008; Quintero et al., 2018; Lenzi et al., 2019), and mechanical sensors, e.g., load cells, position, velocity and current sensors (Huang et al., 2011; Spanias et al., 2018; Khademi and Simon, 2021). However, such sensors can be energy demanding and feature latency in signal outputs for matching human motion resulting in compromised autonomous adaptation (Zhang et al., 2019). On the other hand, surface electromyograms (sEMG) can be used in the assistive robotic system controllers (Huang et al., 2016; Ferris and Schlink, 2017; Spanias et al., 2018; Li et al., 2019; Hosseini et al., 2020; Yin et al., 2020; Hunt et al., 2021; Kyeong et al., 2022; Zhang et al., 2023) gait classification (Huang et al., 2009; Varol et al., 2010; Miller et al., 2013; Liu et al., 2017; Khademi and Simon, 2021) and predicting lower limb joint kinetics and kinematics (Sepulveda et al., 1993; Zhang et al., 2012; Chen et al., 2018; Jephil et al., 2020; Zabre-Gonzalez et al., 2021), which serve well the purpose of the autonomous adaptation. Asif et al. (2021) and Fleming et al. (2021) reported that biomimetic models, artificial neural networks, and support vector machines are widely used for such applications. However, there are several issues to take into account in using sEMG for such purpose one related to preprocessing or avoiding of that, and two related to the number of sensors to be utilized.

First, sEMG signals have complicated and random patterns, making real-time analysis difficult (Reaz et al., 2006; Chowdhury et al., 2013). Halaki and Ginn (2012) reported that amplitude normalization can make interpretation of raw sEMG more accurate for detecting muscle activation. However, the implementation of a real-time normalization imposes additional difficulties in determining the reference value and leads to an elevated computational cost as the necessary periodic calibration of such reference can take several minutes (Tanaka et al., 2022) and disrupt real-time use. Fluctuations across sEMG channels caused by muscle fatigue and variability in skin impedance can also obscure the reference value over longer periods of time (Scheme and Englehart, 2011; Khushaba et al., 2014; Triwiyanto et al., 2018; Ameri et al., 2020) and make sEMG normalization unsuitable for real-time processing (Tanaka et al., 2022). One possibility could be finding static reference values for each targeted muscle through some pre-testing such as a few minutes of stable walking at a certain condition and normalizing the sEMG signals to those references in the real-time implementation. In the amputee prosthesis users, this could be utilized before adding any control to the device provided that it can work passively. This can serve limiting the computation cost of normalization, which needs to be tested in new studies. However, prolonged walking has been shown to cause muscle fatigue (Chambers et al., 2019) and consequently decreased sEMG amplitudes (Schlink et al., 2021). More importantly, studies have shown variations in sEMG amplitudes with changes in walking speed (den Otter et al., 2004), which may compromise accuracy of static reference values. Currently, the use of nonnormalized sEMG supported by feature extraction implementation is the plausible method to be utilized (Fleming et al., 2021). With the aim of achieving autonomous adaptation to improve the human-machine coordination performance in exoskeletons, Ma et al. (2020) used time-domain features of sEMG of eight leg muscles collected from healthy subjects in order to estimate the knee joint angle by using a long short-term memory structure (LSTM). A similar approach was used by Foroutannia et al. (2022) to develop an LSTM architecture that utilizes time-domain features of leg muscles in the prediction of hip joint kinematics.

Second, sEMG signals can be contaminated by noise caused by cross talk and skin conductivity change, or artifacts originating at the skin-electrode interface, signal amplifiers, and external sources (De Luca et al., 2010). Therefore, using a large number of sEMG sensors may cause processing complexity (Hussain et al., 2020), complications in sensor setup design, and impose difficulty in daily use (Fleming et al., 2021). In different studies, the number of sEMG amplitudes utilized for predicting joint kinematics and kinetics varies from eleven (e.g., Huang et al., 2009, 2011; Du et al., 2013; Hargrove et al., 2013; Young et al., 2014; Spanias et al., 2016; Liu et al., 2017) to four (e.g., Hoover et al., 2012; Spanias et al., 2018). Zaffir et al. (2021) developed and compared different types of neural network models for estimating dorsiflexion for robotic ankle-foot orthoses with similar concerns of minimizing the number of muscle inputs and eliminating mechanical sensors. They utilized sEMG data of four leg muscles of healthy participants. Extracted time-domain features were used as inputs, and the best performance was shown by the LSTM neural network.

Third, the original cause of the amputation, the choice of surgical technique and factors such as residual limb length, shape, and subsequent muscle atrophy can affect muscle activity and, consequently, sEMG signals (Isakov et al., 1996; Smith, 2004; Tintle et al., 2010; Ranz et al., 2017). In transtibial amputation, the residual limb muscle availability gets compromised if the amputation is performed closer to the knee (Huang and Ferris, 2012), whereas most muscle mass remains intact in ankle disarticulation (Isaacs-Itua and Sedki, 2018).

Therefore, minimizing the number of sEMG muscle sources utilized in a joint kinematics and kinetics prediction algorithm will make the controller of a powered ankle prosthesis *economic*, and limiting the use of lower leg muscles will make it *practical* for both ankle disarticulation and transtibial amputation. Recently, we developed neural network-based algorithms to predict ankle position and moment using all combinations of sEMG amplitudes of several lower leg muscles (Keleş and Yucesoy, 2020). This approach methodologically paved the way for achieving a user specific algorithm development procedure as the most suitable sEMG variations that either minimizes the total number of sensors or those of lover leg muscles could be identified. However, the use of normalized sEMG exclusively impedes a real-time applicability. In addition, the number of available muscles limited to five restricts a versatile applicability, given the inter-individual differences of potential users. In the present study, the methodology is extended for competency by (i) utilizing nonnormalized sEMG signals coupled with feature extraction and (ii) making muscle input selection comprehensive via the usage of eight lower leg muscles.

The aims were to (1) implement our extended methodology for developing algorithms that predict sagittal ankle kinematics and kinetics during level walking using nonnormalized lower limb sEMG signals, and (2) rank the extensive range of muscle combination variations according to their success to facilitate a user specific selection of sensor inputs for economic and practical control algorithms that can be used in powered ankle prostheses.

## 2. Methods

### 2.1. Data utilized and summary of data collection procedures

Gait data are required for developing, training, and testing neural networks. For that purpose, sEMG amplitudes, sagittal ankle angle, and moment of level walking trials were acquired from the open access data by Lencioni et al. (2019). The data includes (I) trials of level walking at different speeds (0.3–2.3 m/s) collected from fifty able-bodied subjects (25 males, 25 females, age range, 6–72 years, body mass: 18.2–110 kg, body height: 116.6–187.5 cm) and (II) in addition to joint kinematics and kinetics data also nonnormalized sEMG data of tibialis anterior (TA), soleus (SO), medial gastrocnemius (MG), peroneus longus (PL), rectus femoris (RF), vastus medialis (VM), biceps femoris (BF) and gluteus maximus (GMax), which were recorded simultaneously with motion capturing during the level walking trials. Ankle joint kinematics and kinetics data were recorded using a 9-camera motion capture system (SMART system, BTS, Garbagnate Milanese, Italy), total-body LAMB marker set and two force plates (Kistler, Winterthur, Switzerland). The sEMG amplitudes were recorded using an 8-channel wireless EMG recording system (ZeroWirePlus, Cometa, Bareggio, Italy) unilaterally on the dominant side with electrode locations in agreement with SENIAM (Hermens et al., 2000), using pre-amplified self-adhesive Ag-AgCl electrodes (Medtronic Kendall, USA, 24 mm electrode diameter, 10 mm active part diameter, bipolar configuration, 20 mm inter-electrode distance). sEMG amplitudes were band-pass filtered (10 Hz – 400 Hz) before sampling to reduce the aliasing effect, but not normalized. Synchronous data acquisition was managed by the proprietary software of the motion capture system.

In each experiment, the subjects were equipped with the LAMB total-body marker set, which includes retro-reflective markers (12 mm diameter) on lower limbs. As required by the LAMB protocol, additional markers were placed on the medial part of the lower limbs for the preliminary static calibration trial and were removed during the dynamic trials. Subjects were required to wear tight clothes or swimsuits and markers were attached on the skin above bony landmarks with double-sided adhesive tape.

For the level-ground walking task, subjects were initially asked to walk five trials at their natural speed. Then, they were asked to perform the following ten trials while progressively increasing (first 5 trials) or decreasing (latter 5 trials) their speed. No precise indications about gait speed or cadence in order not to induce gait alterations were given.

### 2.2. Neural network development

**Figure 1:**
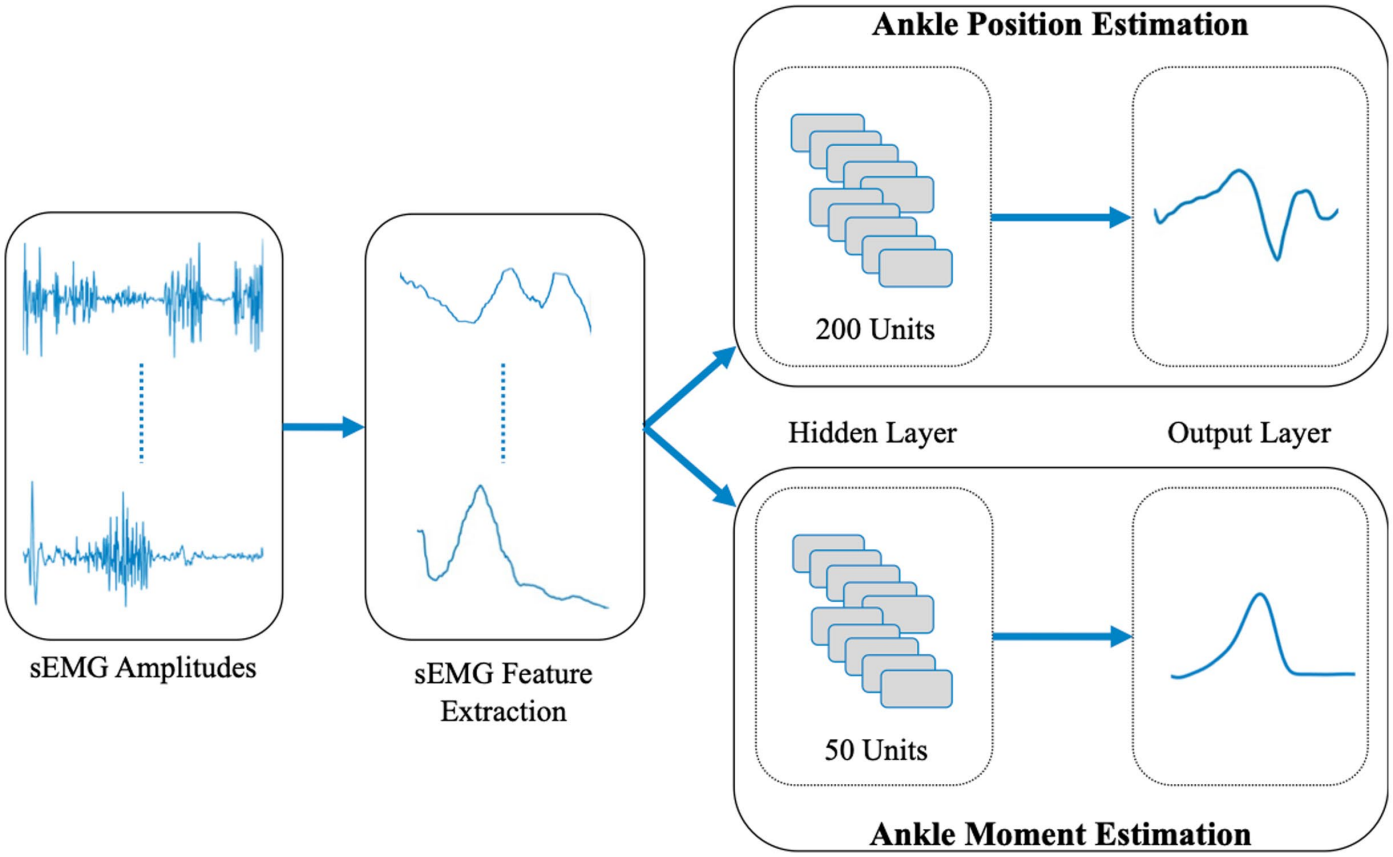
Block diagram of the LSTM structures for ankle position and moment prediction.

All neural networks to be developed feature the following: (1) The long-short term memory (LSTM) neural network architecture was used. LSTM is a deep learning method that is a form of the recurrent neural network model that can make predictions for time series in real-time (Kaushik et al., 2020; Yi et al., 2021). This architecture has been shown to be highly efficient in sEMG-based human motion pattern recognition for identifying the user’s movement (Song et al., 2020). (2) sEMG features, calculated over sEMG amplitudes of leg muscles, were used as LSTM inputs to predict ankle position and moment (see Figure 1 for a block diagram). A similar input integration into LSTM has been shown to be highly efficient in estimating joint angles (Ma et al., 2020; Zaffir et al., 2021; Foroutannia et al., 2022). Presently, two separate LSTMs were developed and trained for predicting ankle joint position and moment. The reason for not using a single LSTM with multiple outputs was (i) to reduce complexity so that LSTMs can be trained and tuned efficiently (Kendall et al., 2018; Crawshaw et al., 2020), (ii) to avoid possible overfitting, which can cause inaccurate predictions for new data (Rebuffi et al., 2017; Zhang and Yang, 2021) and (iii) to achieve a better computational performance (Rebuffi et al., 2017).

#### 2.2.1. sEMG feature selection

sEMG features have been used widely as inputs for sEMG-based robotic controllers (Hudgins et al., 1993; Chan and Green, 2007; Angkoon et al., 2009). Presently, five time-domain sEMG features that can be used efficiently in real-time applications (Phinyomark et al., 2011) were tested and evaluated, which are: *integrated EMG (IEMG), mean absolute value (MAV), Willison amplitude (WAMP), root mean square (RMS)* and *waveform length (WL)*. Their mathematical definitions are presented in Table 1. Since the window size to calculate sEMG features should be less than 300 ms for real-time implementation (Phinyomark et al., 2011), the window size was selected as 150 ms (Huang et al., 2011). To test the success of sEMG features, all possible combinations (in total 31) were studied: 5 single features (e.g., IEMG), 10 two feature combinations (e.g., IEMG+MAV), 10 three feature combinations (e.g., IEMG+MAV + WAMP), 5 four feature combinations (e.g., IEMG+MAV + WAMP+RMS) and 1 five feature combination (i.e., IEMG+MAV + WAMP+RMS + WL). In order to determine the best performing sEMG feature variation, sEMG amplitudes of the muscle combination MG + BF + GMax were used based on the success of this muscle combination in ankle position and moment predictions (Keleş and Yucesoy, 2020).

**Table 1:** Mathematical definition of the sEMG feature extraction methods.

Integrated EMG (IEMG)	$\mathrm{IEMG}=\overset{N}{\underset{n=1}{\sum }}|x_{n}|$
Mean Absolute Value (MAV)	$\mathrm{MAV}=\frac{1}{N}\overset{N}{\underset{n=1}{\sum }}|x_{n}|$
Root Mean Square (RMS)	$\mathrm{RMS}=\sqrt{\frac{1}{N}\overset{N}{\underset{n=1}{\sum }}x_{n}^{2}}$
Waveform Length (WL)	$\mathrm{WL}=\overset{N-1}{\underset{n=1}{\sum }}|x_{n+1}-x_{n}|$
Willison Amplitude (WAMP)	$\mathrm{WAMP}=\overset{N-1}{\underset{n=1}{\sum }}f\left(|x_{n}-x_{n+1}|\right)$ $f\left(x\right)=\{\begin{array}{c} 1,if\;x\geq threshold\\ 0,otherwise \end{array}$

x_n_ represents the sEMG signal in a segment n. N denotes the length of the sEMG signal.

IEMG: sum of the absolute values of the signal in a sliding window. MAV: mean of the absolute values of the signal in a sliding window. RMS: square root of the average power of the sEMG amplitude in a sliding window. WL: the cumulative length of the sEMG amplitude within a sliding window. WAMP: total number of stages resulting from amplitude change between two adjacent points that exceed a defined threshold in a sliding window. Threshold value for WAMP is chosen as 50 mV (Phinyomark et al., 2011).

#### 2.2.2. Muscle selection

sEMG amplitudes of the eight lower extremity muscles included in the dataset were used: the TA, SO, MG, and PL in the lower leg and the RF, BF, VM, and GMax in the upper leg. To test the success of the use of sEMG amplitudes in neural networks inputs for the prediction of ankle position and moment, all possible combinations (in total 255) were studied: 8 single muscles (e.g., TA), 28 two muscle combinations (e.g., TA + SO), 56 three muscle combinations (e.g., TA + SO+MG), 70 four muscle combinations (e.g., TA + SO+MG + PL), 56 five muscle combinations (e.g., TA + SO+MG + PL + RF), 28 six muscle combinations (e.g., TA + SO+MG + PL + RF + BF), 8 seven muscle combinations (e.g., TA + SO+MG + PL + RF + BF + VM), and 1 eight muscle combination (i.e., TA + SO+MG + PL + RF + BF + VM + GMax).

The best performing variation including the minimum total number of muscle inputs is referred to as the *economic variation*. The variation performing better than the economic variation and including the minimum number of lower leg muscle inputs is referred to as the *flexible variation*. The best performing variation including the minimum total number of only upper leg muscles is classified as *practical variation*.

### 2.3. Neural network training

**Figure 2:**
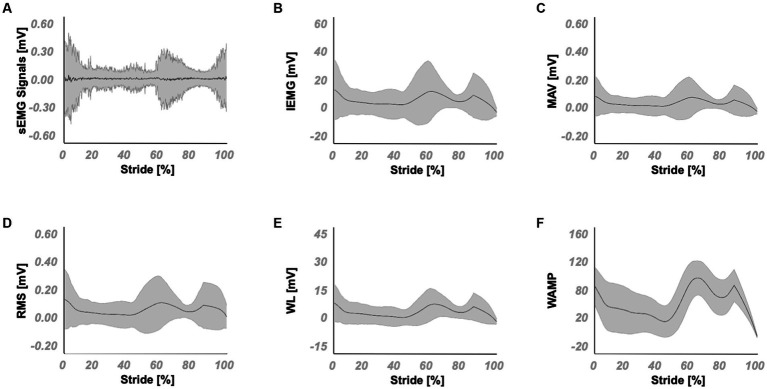
Mean and standard deviation of sEMG signals and its extracted features for the TA muscle. **(A)** the sEMG signals and the corresponding **(B)** IEMG, **(C)** MAV, **(D)** RMS, **(E)** WL and **(F)** WAMP features.

The open access data (Lencioni et al., 2019) contains 844 level walking trials at various walking speeds. Randomly selected 675 datasets (80% of the total) were classified and used as training datasets, and the rest were classified and used as validation datasets. Both training and validation datasets contain the same range of walking speed data (0.3–2.3 m/s) which enabled the LSTMs to be trained and tested for a wide range of walking speeds. The feature extraction function outputs for the TA muscle are exemplified in Figure 2 (see Supplementary Document 1 for the remainder of the muscles). For each muscle within each dataset, the features were extracted and were used in the training and validation of LSTMs developed, which was done separately for ankle joint position and moment predictions. LSTMs were trained within MATLAB, using the Adam optimizer because of its computational efficiency and its success in non-stationary data (Kingma and Ba, 2015). Trainings were stopped after 200 epochs (e.g., Zhang and Ma, 2023). The selection of the number of hidden units for joint position and moment predictor LSTMs was based on previous studies aiming at utilization of sEMG in joint kinetics and kinematics predictions (Kim et al., 2020; Bao et al., 2021), such that a range of 250 units was chosen for both. 200 hidden units for joint position and 50 hidden units for joint moment predictor LSTMs were determined (Figure 1) as the outcome of a systematical variation of the number of hidden units within this range to yield the highest correlations for ankle position and moment changes in the sagittal plane.

### 2.4. Evaluation of the developed neural networks

Normal Q-Q plots for the residuals between original data and LSTM response were used to illustrate if the residuals follow a normal distribution. Two-way ANOVA for repeated measures (factors: %GC and muscle combination) was performed separately for joint position and moment predictions based on correlation coefficients. If significant main effects were found, Bonferroni *post hoc* tests were performed to further locate significant within-factor differences. One-way ANOVA was further used for temporal success assessment based on one-dimensional statistical parametric mapping (SPM) across the gait cycle (GC) using F-statistics (Pataky, 2010). Differences between the original data and estimated outputs were considered significant at *p* < 0.05.

To evaluate the outcome of the developed sEMG feature and muscle combinations (i.e., the best performing one out of all possible 31 sEMG feature combinations x all 255 possible muscle combinations) for joint position and moment predictions, the following were used: (1) Pearson’s correlation coefficient (r) between the original data and LSTM response was calculated. r = 0.90 was selected as a strong correlation threshold. A *miscorrelation score* was defined as the sum of deviation of correlation values from one. (2) Root-mean-square errors (RMSE) between the original data and LSTM response were calculated. An *RMSE score* was defined as the mean of position and moment RMSE values normalized to their respective peak value among all sEMG feature or muscle combinations. (3) SPM analysis was conducted to localize significantly different %GC points between the original data and LSTM response, total number of which are expressed as a percentage of the entire GC. An *SPM Score* was defined as the mean of those percentages for ankle position and moment predictions. (4) An *overall error score* was defined as the product of miscorrelation, RMSE, and SPM scores.

## 3. Results

### 3.1. sEMG feature selection

Table 2 shows the correlation coefficients, miscorrelation scores, RMSE values, RMSE scores, SPM results, and SPM scores of all sEMG feature combinations calculated over sEMG amplitudes of MG + BF + GMax and provides their ranking based on their overall error score.

**Table 2:** The comparison of the performance of sEMG feature variations utilizing the muscle combination MG + BF + GMax.

Rank	sEMG feature variations	Position correlation [r]	Moment correlation [r]	Position RMSE [deg]	Moment RMSE [Nm/kg]	Position SPM [%GC]	Moment SPM [%GC]	Miscorrelation score	Error score	SPM score	Overall error score
1	IEMG+WL	0.9006 ± 0.0831	0.9742 ± 0.0503	4.6781 ± 1.7030	0.1094 ± 0.0610	0.1000	0.0000	0.0626	0.8670	0.0500	0.0027
2	IEMG+MAV + RMS + WAMP	0.8948 ± 0.0879	0.9718 ± 0.0419	4.8151 ± 1.7124	0.1158 ± 0.0554	0.0600	0.1200	0.0667	0.9046	0.0900	0.0054
3	MAV + RMS + WAMP	0.8933 ± 0.1009	0.9732 ± 0.0406	4.8809 ± 1.6684	0.1129 ± 0.0478	0.0600	0.2500	0.0667	0.8998	0.1550	0.0093
4	IEMG+RMS + WL	0.9031 ± 0.1034	0.9710 ± 0.0750	4.6766 ± 1.7467	0.1154 ± 0.0725	0.0300	0.3300	0.0629	0.8898	0.1800	0.0101
5	IEMG	0.8955 ± 0.0848	0.9780 ± 0.0250	4.9175 ± 1.8937	0.1090 ± 0.0470	0.1500	0.2200	0.0633	0.8883	0.1850	0.0104
6	IEMG+MAV + RMS + WL	0.9095 ± 0.0785	0.9747 ± 0.0435	4.6147 ± 1.5559	0.1096 ± 0.0564	0.2000	0.2500	0.0579	0.8617	0.2250	0.0112
7	IEMG+MAV + WL	0.8962 ± 0.1013	0.9758 ± 0.0369	4.7377 ± 1.7184	0.1083 ± 0.0533	0.2100	0.2000	0.0640	0.8684	0.2050	0.0114
8	IEMG+RMS + WAMP	0.8940 ± 0.1038	0.9727 ± 0.0347	4.6181 ± 1.7669	0.1160 ± 0.0567	0.1400	0.2500	0.0667	0.8865	0.1950	0.0115
9	WAMP	0.8884 ± 0.0925	0.9763 ± 0.0263	4.9206 ± 1.6076	0.1109 ± 0.0447	0.1800	0.2200	0.0676	0.8959	0.2000	0.0121
10	RMS	0.8888 ± 0.0955	0.9757 ± 0.0255	5.0877 ± 2.0147	0.1142 ± 0.0460	0.1500	0.2700	0.0677	0.9245	0.2100	0.0132
11	WL	0.9006 ± 0.0830	0.9814 ± 0.0209	4.8743 ± 1.7730	0.1020 ± 0.0419	0.2400	0.3200	0.0590	0.8574	0.2800	0.0142
12	MAV + WAMP+WL	0.8860 ± 0.1081	0.9734 ± 0.0342	4.9387 ± 1.8112	0.1154 ± 0.0550	0.3200	0.1300	0.0703	0.9149	0.2250	0.0145
13	RMS + WAMP+WL	0.8987 ± 0.0965	0.9706 ± 0.0779	4.7733 ± 1.5983	0.1145 ± 0.0575	0.1900	0.3100	0.0653	0.8956	0.2500	0.0146
14	IEMG+MAV + RMS	0.9034 ± 0.0733	0.9800 ± 0.0269	4.7612 ± 1.6848	0.1076 ± 0.0467	0.0300	0.6100	0.0583	0.8680	0.3200	0.0162
15	RMS + WL	0.9023 ± 0.0742	0.9782 ± 0.0329	4.8670 ± 1.6755	0.1062 ± 0.0552	0.2300	0.4000	0.0597	0.8728	0.3150	0.0164
16	MAV + RMS + WL	0.8961 ± 0.1182	0.9671 ± 0.1330	4.8736 ± 1.8146	0.1122 ± 0.0724	0.3400	0.2000	0.0684	0.8964	0.2700	0.0166
17	MAV	0.8824 ± 0.1078	0.9758 ± 0.0251	5.1429 ± 2.0960	0.1147 ± 0.0462	0.1900	0.3200	0.0709	0.9317	0.2550	0.0168
18	IEMG+MAV	0.8885 ± 0.0885	0.9745 ± 0.0408	5.0225 ± 1.8550	0.1142 ± 0.0575	0.2000	0.3500	0.0685	0.9183	0.2750	0.0173
19	IEMG+MAV + RMS + WAMP+WL	0.9006 ± 0.1162	0.9663 ± 0.0627	4.5362 ± 1.8213	0.1204 ± 0.0689	0.1100	0.4900	0.0665	0.8956	0.3000	0.0179
20	WAMP+WL	0.8927 ± 0.0971	0.9665 ± 0.0743	4.7689 ± 1.6955	0.1196 ± 0.0634	0.0800	0.5600	0.0704	0.9148	0.3200	0.0206
21	MAV + RMS + WAMP+WL	0.8965 ± 0.0780	0.9567 ± 0.1015	4.8439 ± 1.6630	0.1252 ± 0.0792	0.1000	0.5400	0.0734	0.9434	0.3200	0.0222
22	RMS + WAMP	0.8776 ± 0.1066	0.9730 ± 0.0361	5.0264 ± 1.8036	0.1168 ± 0.0514	0.3900	0.2600	0.0747	0.9287	0.3250	0.0225
23	MAV + RMS	0.8765 ± 0.1068	0.9766 ± 0.0241	5.2271 ± 2.0433	0.1132 ± 0.0451	0.3000	0.4000	0.0734	0.9340	0.3500	0.0240
24	IEMG+MAV + WAMP+WL	0.8935 ± 0.1044	0.9605 ± 0.0849	4.6255 ± 1.6296	0.1275 ± 0.0670	0.1400	0.5700	0.0730	0.9313	0.3550	0.0241
25	IEMG+RMS	0.8952 ± 0.0880	0.9744 ± 0.0349	4.9570 ± 1.8372	0.1120 ± 0.0518	0.1800	0.6800	0.0652	0.9036	0.4300	0.0253
26	IEMG+RMS + WAMP+WL	0.8910 ± 0.0998	0.9709 ± 0.0290	4.8587 ± 1.8341	0.1202 ± 0.0493	0.6100	0.2100	0.0691	0.9257	0.4100	0.0262
27	IEMG+WAMP	0.8751 ± 0.1106	0.9705 ± 0.0461	5.0310 ± 1.9238	0.1155 ± 0.0601	0.5500	0.2200	0.0772	0.9241	0.3850	0.0275
28	MAV + WAMP	0.8827 ± 0.1034	0.9766 ± 0.0236	4.9713 ± 1.6828	0.1144 ± 0.0446	0.3600	0.5200	0.0703	0.9142	0.4400	0.0283
29	IEMG+WAMP+WL	0.8906 ± 0.1151	0.9619 ± 0.1234	4.8330 ± 1.7555	0.1214 ± 0.0713	0.6200	0.2300	0.0737	0.9278	0.4250	0.0291
30	MAV + WL	0.8924 ± 0.1383	0.9796 ± 0.0244	4.9329 ± 1.8115	0.1064 ± 0.0463	0.5900	0.5800	0.0640	0.8798	0.5850	0.0329
31	IEMG+MAV + WAMP	0.8768 ± 0.1085	0.9611 ± 0.0591	4.9993 ± 1.9492	0.1304 ± 0.0677	0.4800	0.5900	0.0811	0.9782	0.5350	0.0424

Grey shaded cells show the feature variations that do not provide strongly correlated position and moment predictions (i.e., *r* < 0.90). IEMG, integrated EMG; MAV, mean absolute value; WAMP, Willison amplitude; RMS, root mean square; WL, waveform length.

The five feature combination (IEMG+MAV + RMS + WAMP+WL) shows a strong correlation (*r*_position_ = 0.9006 and *r*_moment_ = 0.9663) and ranks 19th amongst all.

Four feature combinations: Only IEMG+MAV + RMS + WL shows a strong correlation (*r*_position_ = 0.9095 and *r*_moment_ = 0.9747) and ranks 6^th^ amongst all.

Three feature combinations: 2 of 10 variations show strong correlations (*r*_position_ > 0.90 and *r*_moment_ > 0.97). IEMG+RMS + WL shows the strongest correlation (*r*_position_ = 0.9031 and *r*_moment_ = 0.9710) and ranks 4^th^ amongst all variations.

Two feature combinations: 2 of 10 variations show strong correlations (*r*_position_ > 0.90 and *r*_moment_ > 0.97). IEMG+WL shows the strongest correlation (*r*_position_ = 0.9006 and *r*_moment_ = 0.9742) and ranks 1st amongst all variations.

Single features: Only WL shows a strong correlation (*r*_position_ = 0.9006 and *r*_moment_ = 0.9814) and ranks 11th amongst all.

**Figure 3:**
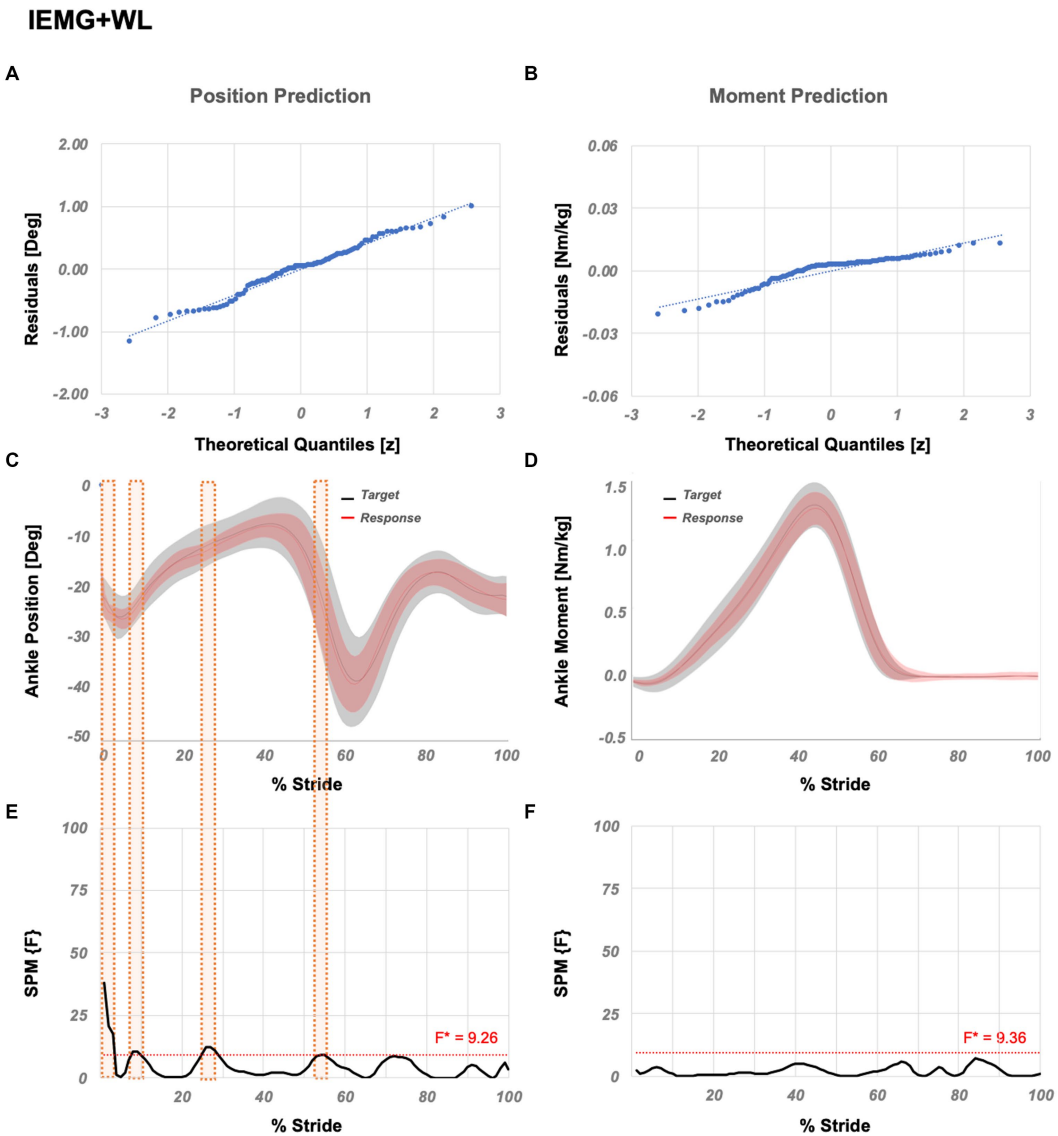
Temporal success assessment for IEMG+WL. The normal Q-Q plots for the residuals between original data and LSTM response for ankle **(A)** position and **(B)** moment. Mean and standard deviation of estimated NN response vs. original data for ankle **(C)** position and **(D)** moment, as a function of % stride. Statistical parametric mapping using *F*-statistics (SPM{F}) for ankle **(E)** position and **(F)** moment as a function of % stride shows values below the threshold (i.e., for *p* < 0.05, F*position = 9.26, F*moment = 9.36). IEMG abbreviates integrated EMG and WL abbreviates waveform length.

Results show that IEMG+WL yields the best position and moment prediction performance. SPM analysis of the IEMG+WL (Figure 3) showed that significant localized differences between the actual and estimated outputs are limited to 10 points (1–3%, 8–9%, 25–28%, and 54% GC) for ankle position, whereas no significantly different points were localized for ankle moment.

### 3.2. Muscle selection

ANOVA (factors: %GC and muscle combination) showed significant main effects on joint position predictions, but no interaction. ANOVA (factors: %GC and muscle combination) showed significant main effects on joint moment predictions and a significant interaction. *Post hoc* testing showed significant effects of muscle combination based on comparisons of correlation coefficients for (i) 239 out of 465 combinations for joint position predictions, and (ii) 227 out of 465 combinations for joint moment predictions.

Table 3 shows the correlation coefficients, miscorrelation scores, RMSE values, RMSE scores, SPM results, and SPM scores of best performing 30 muscle combinations determined utilizing the sEMG feature IEMG+WL followed by selected exclusively upper leg muscle combinations, and provides their ranking based on their overall error score (see Supplementary Document 2 for all variations).

**Table 3:** The best performing 30 muscle combinations determined by utilizing the sEMG feature IEMG+WL.

Rank	Muscle variations	Position correlation [r]	Moment correlation [r]	Position RMSE [deg]	Moment RMSE [Nm/kg]	Position SPM [%GC]	Moment SPM [%GC]	Miscorrelation score	Error score	SPM score	Overall error score
1	MG + RF + VM ^†^	0.9099 ± 0.0711	0.9707 ± 0.0784	4.5923 ± 1.4815	0.1072 ± 0.0660	0.0300	0.0400	0.0597	0.8819	0.0350	0.0018
2	GMax+RF + SO	0.8978 ± 0.0884	0.9716 ± 0.0400	4.8759 ± 1.6262	0.1151 ± 0.0515	0.0500	0.0100	0.0653	0.9415	0.0300	0.0018
3	BF + MG + GMax+PL + RF + SO ^†^	0.9134 ± 0.0938	0.9714 ± 0.0600	4.3291 ± 1.6537	0.1067 ± 0.0624	0.0700	0.0200	0.0576	0.8543	0.0450	0.0022
4	BF + MG + GMax+RF + SO+TA + VM ^†^	0.9246 ± 0.0614	0.9803 ± 0.0270	4.1877 ± 1.5685	0.1012 ± 0.0514	0.1200	0.0000	0.0475	0.8182	0.0600	0.0023
5	MG + GMax+PL + VM ^†^	0.9101 ± 0.0793	0.9725 ± 0.0753	4.5901 ± 1.6014	0.1085 ± 0.0601	0.1000	0.0000	0.0587	0.8869	0.0500	0.0026
6	BF + MG + GMax ^* †^	0.9006 ± 0.0831	0.9742 ± 0.0503	4.6781 ± 1.7030	0.1094 ± 0.0610	0.1000	0.0000	0.0626	0.8991	0.0500	0.0028
7	BF + MG + GMax+PL + RF + SO+TA + VM ^* †^	0.9205 ± 0.0788	0.9695 ± 0.0862	4.2841 ± 1.6202	0.1060 ± 0.0669	0.0100	0.1200	0.0550	0.8471	0.0650	0.0030
8	MG + GMax+RF + TA ^†^	0.9123 ± 0.0730	0.9789 ± 0.0331	4.5078 ± 1.6104	0.1034 ± 0.0551	0.1100	0.0300	0.0544	0.8582	0.0700	0.0033
9	MG + GMax+PL + RF + SO+TA ^†^	0.9267 ± 0.0548	0.9733 ± 0.0490	4.1133 ± 1.4718	0.1079 ± 0.0573	0.0500	0.1100	0.0500	0.8384	0.0800	0.0034
10	MG + GMax+TA ^* †^	0.9164 ± 0.0670	0.9782 ± 0.0276	4.4843 ± 1.5643	0.1059 ± 0.0538	0.1200	0.0300	0.0527	0.8661	0.0750	0.0034
11	RF + TA ^* †^	0.9174 ± 0.0689	0.9776 ± 0.0238	4.6396 ± 1.5971	0.1081 ± 0.0439	0.0300	0.1200	0.0525	0.8901	0.0750	0.0035
12	MG + SO+TA ^* †^	0.9197 ± 0.0587	0.9769 ± 0.0373	4.4188 ± 1.6482	0.1072 ± 0.0529	0.1100	0.0500	0.0517	0.8651	0.0800	0.0036
13	BF + MG + GMax+PL + SO+TA ^†^	0.9166 ± 0.0938	0.9760 ± 0.0529	4.3324 ± 1.6361	0.1070 ± 0.0595	0.0200	0.1400	0.0537	0.8559	0.0800	0.0037
14	BF + PL + RF + TA ^†^	0.9161 ± 0.0757	0.9734 ± 0.0364	4.4649 ± 1.5828	0.1119 ± 0.0496	0.1000	0.0500	0.0552	0.8887	0.0750	0.0037
15	PL + SO+TA + VM ^* †^	0.9216 ± 0.0615	0.9768 ± 0.0291	4.4417 ± 1.4206	0.1104 ± 0.0500	0.1200	0.0600	0.0508	0.8803	0.0900	0.0040
16	MG + PL + TA ^* †^	0.9203 ± 0.0732	0.9757 ± 0.0498	4.3252 ± 1.5455	0.1083 ± 0.0599	0.1200	0.0700	0.0520	0.8605	0.0950	0.0043
17	MG + GMax+PL + RF + TA ^†^	0.9099 ± 0.1285	0.9779 ± 0.0290	4.3088 ± 1.6608	0.1068 ± 0.0500	0.1200	0.0600	0.0561	0.8528	0.0900	0.0043
18	BF+ MG + RF + SO+TA + VM ^†^	0.9169 ± 0.0831	0.9754 ± 0.0553	4.2226 ± 1.6019	0.1048 ± 0.0536	0.1400	0.0600	0.0539	0.8363	0.1000	0.0045
19	MG + GMax+PL + RF + SO+VM ^*^	0.9136 ± 0.0699	0.9734 ± 0.0476	4.5057 ± 1.4465	0.1078 ± 0.0570	0.0900	0.1000	0.0565	0.8759	0.0950	0.0047
20	MG + GMax+RF + SO+TA ^†^	0.9196 ± 0.0735	0.9725 ± 0.0524	4.3227 ± 1.5550	0.1090 ± 0.0558	0.0600	0.1500	0.0539	0.8631	0.1050	0.0049
21	BF + GMax+VM ^†^	0.8904 ± 0.1091	0.9668 ± 0.0646	5.0332 ± 1.7615	0.1212 ± 0.0652	0.1300	0.0100	0.0714	0.9817	0.0700	0.0049
22	GMax+PL + RF ^* †^	0.9068 ± 0.0759	0.9689 ± 0.0859	4.7873 ± 1.6091	0.1143 ± 0.0567	0.1700	0.0000	0.0622	0.9297	0.0850	0.0049
23	BF + MG + PL + RF ^* †^	0.9102 ± 0.1012	0.9692 ± 0.1117	4.5424 ± 1.6870	0.1074 ± 0.0657	0.0800	0.1100	0.0603	0.8778	0.0950	0.0050
24	BF + MG + GMax+PL + VM ^* †^	0.9097 ± 0.0738	0.9788 ± 0.0280	4.4883 ± 1.4887	0.1065 ± 0.0479	0.1000	0.1100	0.0558	0.8689	0.1050	0.0051
25	MG + GMax+RF + SO+VM	0.9119 ± 0.0676	0.9765 ± 0.0326	4.5431 ± 1.6573	0.1079 ± 0.0512	0.1000	0.1100	0.0558	0.8799	0.1050	0.0052
26	BF + RF + TA ^* †^	0.9164 ± 0.0675	0.9744 ± 0.0406	4.5841 ± 1.5756	0.1124 ± 0.0485	0.0600	0.1500	0.0546	0.9023	0.1050	0.0052
27	BF + PL + SO+VM ^* †^	0.9156 ± 0.0641	0.9649 ± 0.0927	4.5507 ± 1.4057	0.1183 ± 0.0742	0.0400	0.1600	0.0597	0.9231	0.1000	0.0055
28	MG + PL + RF + SO+TA ^* †^	0.9212 ± 0.0628	0.9589 ± 0.1556	4.3659 ± 1.4639	0.1113 ± 0.0748	0.1600	0.0500	0.0600	0.8767	0.1050	0.0055
29	BF + MG + GMax+PL + SO+VM ^* †^	0.9213 ± 0.0737	0.9748 ± 0.0422	4.2727 ± 1.5538	0.1077 ± 0.0564	0.0100	0.2400	0.0520	0.8530	0.1250	0.0055
30	BF + MG + VM ^* †^	0.9201 ± 0.0675	0.9769 ± 0.0371	4.4867 ± 1.6031	0.1083 ± 0.0548	0.0300	0.2300	0.0515	0.8761	0.1300	0.0059
106	GMax+VM ^*^	0.9010 ± 0.0805	0.9718 ± 0.0326	4.8927 ± 1.6140	0.1193 ± 0.0501	0.0900	0.2600	0.0636	0.9603	0.1750	0.0107
154	BF + GMax+RF + VM ^* †^	0.9018 ± 0.0924	0.9659 ± 0.0898	4.8817 ± 1.6350	0.1186 ± 0.0637	0.3300	0.1100	0.0662	0.9564	0.2200	0.0139
247	RF + VM ^* †^	0.9009 ± 0.0784	0.9712 ± 0.0451	4.8933 ± 1.6459	0.1181 ± 0.0551	0.3900	0.5800	0.0640	0.9555	0.4850	0.0296

Darker grey shaded cells show muscle variations that do not provide strongly correlated position and moment predictions (i.e., *r* < 0.90). Lighter gray hatched cells exemplify solely upper leg muscle variations that provide strongly correlated position and moment predictions (i.e., *r* > = 0.90). Based on significantly different correlation coefficients [r] (i.e., if any one of the position or moment correlations, or both are significantly different for a muscle variation), ^(*)^ and ^(†)^ indicate significantly different muscle variations from the flexible (MG + RF + VM) and practical (GMax + VM) muscle variations, respectively. TA: tibialis anterior, SO: soleus, MG: medial gastrocnemius, PL: peroneus longus, RF: rectus femoris, BF: biceps femoris, VM: vastus medialis, GMax: gluteus maximus.

The eight muscle combination (BF + MG + GMax+PL + RF + SO+TA + VM) shows strong correlation (*r*_position_ = 0.9205 and *r*_moment_ = 0.9695) and ranks 7th amongst all variations.

Seven muscle combinations: All variations show strong correlations (*r*_position_ > 0.90 and *r*_moment_ > 0.97). BF + MG + GMax+RF + SO+TA + VM shows the strongest correlation and ranks 4^th^ amongst all variations (*r*_position_ = 0.9246 and *r*_moment_ = 0.9803).

Six muscle combinations: 26 of 28 variations show strong correlations (*r*_position_ > 0.90 and *r*_moment_ > 0.95). BF + MG + GMax+PL + RF + SO shows the strongest correlation and ranks 3rd amongst all variations (*r*_position_ = 0.9134 and *r*_moment_ = 0.9714).

Five muscle combinations: All variations show strong correlations (*r*_position_ > 0.90 and *r*_moment_ > 0.95). MG + GMax+PL + RF + TA shows the strongest correlation and ranks 17th amongst all variations (*r*_position_ = 0.9099 and *r*_moment_ = 0.9779).

Four muscle combinations: 66 of 70 variations show strong correlations (*r*_position_ > 0.90 and *r*_moment_ > 0.96). MG + GMax+PL + VM shows the strongest correlation and ranks 5th amongst all variations (*r*_position_ = 0.9101 and *r*_moment_ = 0.9725).

Three muscle combinations: 45 of 56 variations show strong correlations (*r*_position_ > 0.90 and *r*_moment_ > 0.96). MG + RF + VM shows the strongest correlation and ranks 1st amongst all variations (*r*_position_ = 0.9099 and *r*_moment_ = 0.9707).

Two muscle combinations: 21 of 28 variations show strong correlations (*r*_position_ > 0.90 and *r*_moment_ > 0.96). RF + TA shows the strongest correlation and ranks 11th amongst all variations (*r*_position_ = 0.9174 and *r*_moment_ = 0.9776).

Single muscles: 2 of 8 variations show strong correlations (*r*_position_ > 0.90 and *r*_moment_ > 0.97). PL shows the strongest correlation and ranks 69th amongst all variations (*r*_position_ = 0.9001 and *r*_moment_ = 0.9703).

**Figure 4:**
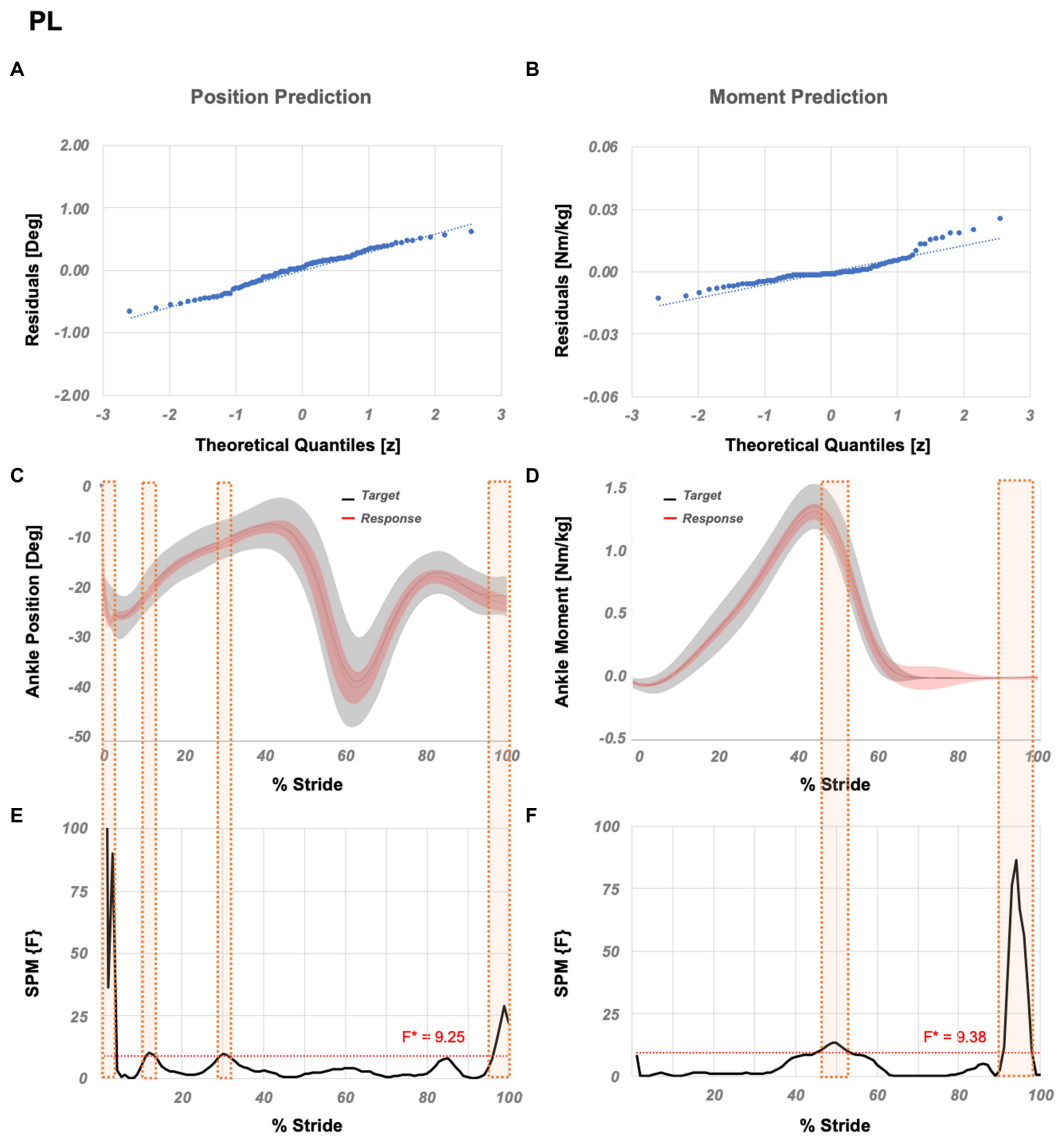
Temporal success assessment for PL. The normal Q-Q plots for the residuals between original data and LSTM response for ankle **(A)** position and **(B)** moment. Mean and standard deviation of estimated NN response vs. original data for ankle **(C)** position and **(D)** moment, as a function of % stride. Statistical parametric mapping using F-statistics (SPM{F}) for ankle **(E)** position and **(F)** moment as a function of % stride shows values below the threshold (i.e., for p < 0.05, F*position = 9.25, F*moment = 9.38). PL abbreviates peroneus longus.

*Economic variation* is PL which ranks 69th amongst all variations. SPM analysis (Figure 4) showed that significant localized differences between the actual and estimated outputs are limited to 1–3%, 12–13%, 30–31%, and 97–100% GC for the position and, 45–53% and 91–98% GC for the moment prediction.

**Figure 5:**
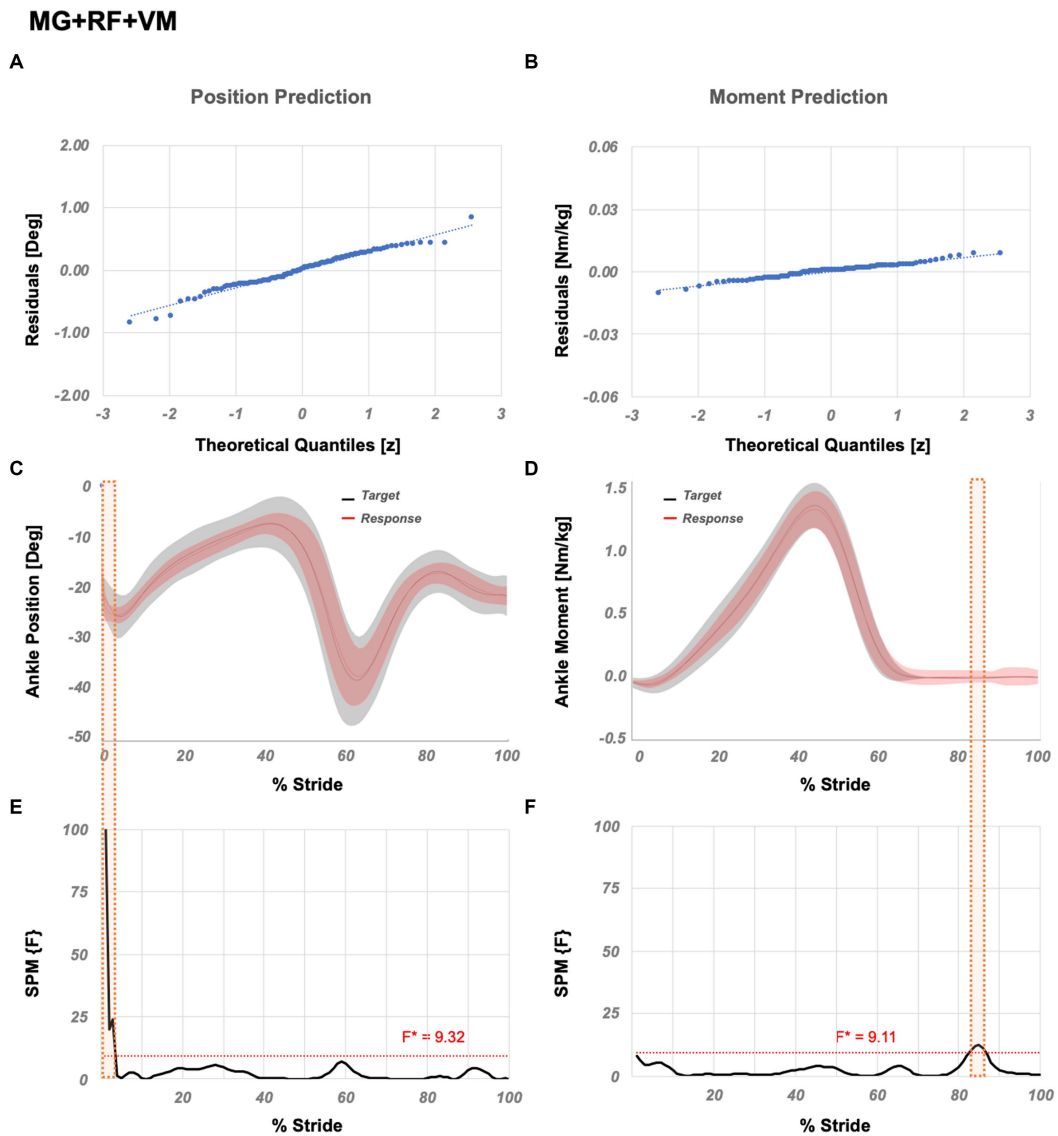
Temporal success assessment for MG + RF + VM. The normal Q-Q plots for the residuals between original data and LSTM response for ankle **(A)** position and **(B)** moment. Mean and standard deviation of estimated NN response vs. original data for ankle **(C)** position and **(D)** moment, as a function of % stride. Statistical parametric mapping using F-statistics (SPM{F}) for ankle **(E)** position and **(F)** moment as a function of % stride shows values below the threshold (i.e., for *p* < 0.05, F*position = 9.32, F*moment = 9.11). MG abbreviates medial gastrocnemius, RF abbreviates rectus femoris, and VM abbreviates vastus medialis.

*Flexible variation* is MG + RF + VM, which ranks the 1st amongst all variations. Table 3. further shows muscle variations with significantly different correlation coefficients compared to MG + RF + VM. SPM analysis (Figure 5) showed that significant localized differences between the actual and estimated outputs occur between 1–3% GC for the position and, 83–86% GC for the moment prediction.

**Figure 6:**
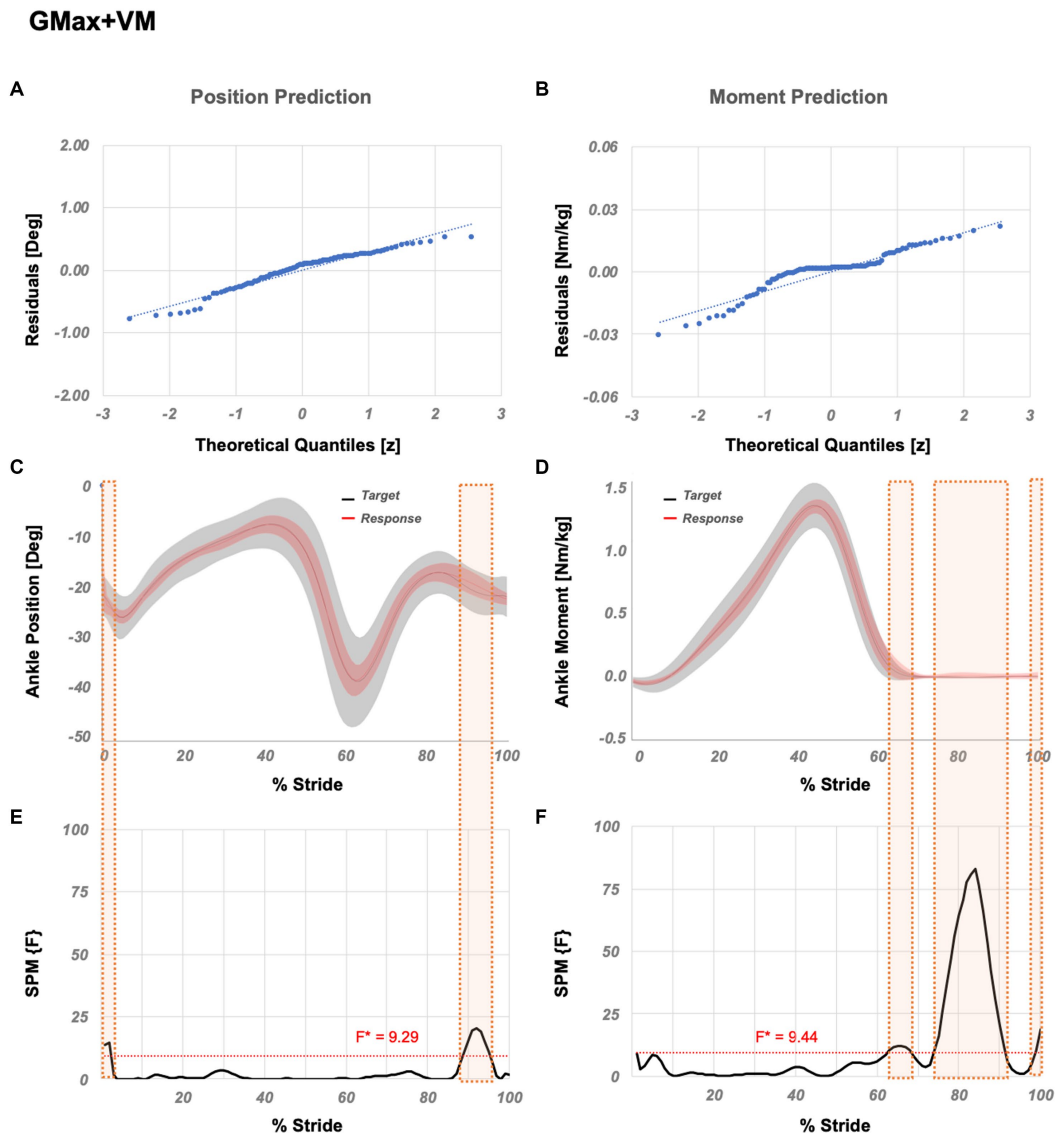
Temporal success assessment for GMax+VM. The normal Q-Q plots for the residuals between original data and LSTM response for ankle **(A)** position and **(B)** moment. Mean and standard deviation of estimated NN response vs. original data for ankle **(C)** position and **(D)** moment, as a function of % stride. Statistical parametric mapping using *F*-statistics (SPM{F}) for ankle **(E)** position and **(F)** moment as a function of % stride shows values below the threshold (i.e., for *p* < 0.05, F*position = 9.29, F*moment = 9.44). GMax abbreviates gluteus maximus and VM abbreviates vastus medialis.

*Practical variation* is GMax+VM, which ranks the 106th amongst all variations. Table 3. further shows muscle variations with significantly different correlation coefficients compared to GMax+VM. SPM analysis (Figure 6) showed that significant localized differences between the actual and estimated outputs occur between 1–2%, and 89–95% GC for the position and 63–68% 74–91%, and 99–100% GC for the moment prediction.

## 4. Discussion

Recent technological improvements in hardware development have advanced the field of lower limb prostheses (Azocar et al., 2018; Elery et al., 2018; Lenzi et al., 2018). However, an advanced autonomous adaptation is still required to achieve seamless and natural ambulation (Fleming et al., 2021). Therefore, studies focused on sEMG-based algorithms for prosthesis controllers have started to accumulate (Dawley et al., 2013; Wang et al., 2013; Huang et al., 2016; Spanias et al., 2018). Recently, we developed neural networks showing that ankle position and moment changes during level walking can be predicted by using normalized sEMG amplitudes of leg muscles (Keleş and Yucesoy, 2020). However, the use of normalized sEMG amplitudes was a limitation which is addressed in the present study.

Souza et al. (2014) indicated that neuromuscular control schemes merging timing-based approaches could advance prosthesis control. Since the action patterns of walking occur in a time series (Song et al., 2020) and sEMG amplitudes show variability in time and are cyclic (Souza et al., 2014), in order to make accurate predictions of joint movement using data from past is essential. LSTM architecture contains a set of memory blocks (Ma et al., 2020) that can maintain its state over time (Greff et al., 2017), enabling the structure to remember the previous inputs (Song et al., 2020). Consequently, LSTM is widely used owing to its capability of generating accurate outputs for time series: the action patterns of walking occur in a time series (Song et al., 2020), and LSTM generates outputs based on the inputs from the past (Greff et al., 2017), making it suitable for predicting joint kinematics and kinetics (Ma et al., 2020). Also, the sEMG feature extraction is used widely, where features are extracted within a sliding window and used as inputs to predict joint kinetics or kinematics (Bi et al., 2019; Gupta et al., 2020; Zhang et al., 2021; Chen et al., 2022; Rabe and Fey, 2022; Truong et al., 2023). Previous studies have shown that using sEMG features as input (e.g., Spanias et al., 2015, 2018), the LSTM structure (e.g., Ren et al., 2022), and their combined implementations (e.g., Song et al., 2020) provide a successful prediction of intended motion. Although prediction of ankle position and moment was not sought after, these studies demonstrated that accurate predictions using time-domain sEMG features and LSTM is plausible. Therefore, an LSTM structure that predicts ankle position and moment during level walking using features extracted from nonnormalized sEMG amplitudes was implemented in the present study. Results showed that the developed LSTMs can predict ankle position and moment during level walking with up to 0.9292 and 0.9813 correlations, respectively. Note that, Huihui et al. (2018) reported an RMSE of 4.80° in ankle position prediction, whereas Hahn and O’Keefe (2008) reported an RMSE of 0.10 Nm/kg in ankle moment prediction. However, those authors utilized 5 and 7 muscles, respectively. Ma et al. (2020) estimated the knee joint angle using 8 muscles with an RMSE of 4.60°, whereas Farmer et al. (2014) estimated the ankle joint angle using 3 lower leg muscles and reported an RMSE of 5.40°. By utilizing only three muscles, the present LSTM structure yielded a compatible success with 4.5923° and 0.1072 Nm/kg RMSE.

Although studies aiming to develop sEMG-based lower limb prosthesis controllers have increased in the last decade, there is still no commercialized product that relies on neuromuscular input (Fleming et al., 2021). Song et al. (2020) predicted the movement patterns using normalized sEMG amplitudes of RF, VM, vastus lateralis, and semitendinosus muscles to extract features within a sliding window size of 1,024 ms. However, a window size less than 300 ms was suggested to support implementation of feature extraction in real-time prosthetic control (Phinyomark et al., 2011). Also, sEMG normalization is a non-specific method in terms of the required data to determine the reference value (Halaki and Ginn, 2012), e.g., the large window size can be a major limitation for the required computation and real-time applicability. Spanias et al. (2015) and Spanias et al. (2018) utilized the same number of neuromuscular input and achieved the use of nonnormalized sEMG amplitudes collected from RF, tensor fasciae latae, semitendinosus, and adductor magnus to extract features within a window size lower than 300 ms. Nonetheless, they have implemented the additional mechanical sensor, which can be energy consuming and could be a trade-off in autonomous adaptation due to its latency in generating the output for the human locomotion (Zhang et al., 2019). Utilizing solely nonnormalized sEMG amplitude was achieved but the number of neuromuscular inputs was increased. In order to recognize the locomotion modes, Huang et al. (2011) utilized sEMG amplitudes of RF, VM, sartorius, vastus lateralis, gracilis, biceps femoris long head, semitendinosus, biceps femoris short head, and adductor magnus muscles. Hargrove et al. (2013) developed a pattern recognition algorithm using sEMG amplitudes of BF, RF, VM, vastus lateralis, sartorius, gracilis, adductor magnus, and tensor fasciae latae. Chen et al. (2018) developed a deep belief network-based algorithm to estimate lower limb joint angles including the ankle and utilized sEMG amplitudes collected from BF, VM, RF, MG, TA, SO, MG, semitendinosus, vastus lateralis, and sartorius. Huihui et al. (2018) implemented the use of feature extraction and nonnormalized sEMG amplitudes collected from the external gastrocnemius, gastrocnemius, SO, TA, and tibialis longus in order to estimate ankle joint angle. Wang et al. (2019) used sEMG amplitudes of RF, VM, BF, semitendinosus, and gastrocnemius to estimate knee joint angles. These studies show that the prosthesis controller can be advanced with several sEMG inputs. However, such implementation may bear difficulties regarding the processing complexity (Hussain et al., 2020). Au et al. (2008) limited the use of neuromuscular input size and utilized TA and gastrocnemius muscles in order to develop a powered ankle prosthesis controller, whereas Au et al. (2005) and Farmer et al. (2014) used the same muscles and included the SO to predict the ankle position. Similarly, Zabre-Gonzalez et al. (2021) developed a neural network-based algorithm to predict ankle position and moment using TA and MG muscles. Jephil et al. (2020) utilized the same muscles with the addition of the lateral gastrocnemius to estimate the ankle joint torque and angle. These studies show that the neuromuscular input size can be reduced by using the lower leg muscles. However, Huang and Ferris (2012) showed that sEMG amplitudes patterns of the TA, gastrocnemius medial head, and gastrocnemius lateral head in transtibial amputees, recorded within the prosthetic socket during walking, have high inter-subject variability. Therefore, limiting lower leg muscle inputs or using solely upper leg muscles can increase the compatibility of the prediction algorithms for different levels of amputations. In the present study, with the goal of real-time applicability, we developed LSTMs to predict ankle position and ankle moment using time-domain features extracted within 150 ms from non-normalized sEMG amplitudes. The proposed LSTM structure eliminates the need for external mechanical sensor inputs which cause high processing complexity. Also, the presented structure is capable of generating strongly correlated predictions by using sEMG amplitudes of GMax and VM muscles, addressing the compatibility issue. Furthermore, none of the previous studies provided a systematic analysis for the selection of muscles to be used as input. We recently developed a methodology to systematically study all possible variations of sEMG inputs from lower leg muscles (Keleş and Yucesoy, 2020). Yet, the implementation of this methodology to level walking was limited to the usage of five muscle inputs (TA, MG, RF, BF, and GMax) and more importantly to normalized sEMG data exclusively. Presently, we extended the systematic analysis by including also SO, PL, and VM muscles. Note that, indeed the PL muscle was shown to be relevant as even a single input, whereas the analyses indicated that VM is important in limiting the usage of lower leg muscles. However, the bigger contribution of the present study was the extension of the methodology to make it suitable for a real-time implementation, which now involves utilization of features extracted from nonnormalized sEMG signals as inputs to the LSTM architecture. Integration of the SPM analysis outcome into the performance metric is also a new approach allowing for a better tracking of specific %GC’s showing significant differences between the original data and LSTM response. These present achievements provide a good versatility by increasing the choices of upper and lower leg muscles and improve the user specific applicability of sEMG-based ankle position and moment prediction procedures in powered ankle prosthesis control algorithms.

The present study shows that the LSTM is suitable for ankle position and moment prediction by using the time-domain features extracted from the nonnormalized sEMG amplitudes as input. Our comprehensive time-domain sEMG feature selection analysis showed that the best performing variation is IEMG+WL, which is frequently implemented in sEMG-based prosthesis controller studies (Fleming et al., 2021) and is feasible to be used in real-time applications (Phinyomark et al., 2011). Presently, we used the muscle combination MG + BF + GMax for sEMG feature selection. Previous studies with a similar aim of utilizing sEMG in predicting joint kinematics and kinetics conducted either a feature (e.g., Phinyomark et al., 2011) or a muscle combination selection (e.g., Wang et al., 2021), or report only the utilized feature per muscle (Phinyomark et al., 2011; Li et al., 2021). Note that, taking into account the challenging nature of determining which specific parameter or combination of parameters is responsible for an improved neural network output (Goodfellow et al., 2016), a separate evaluation of the effects of multiple parameters has been suggested (Molnar, 2020). The complexity of the neural networks can consequently be reduced for an efficient training (Kendall et al., 2018). Our present approach, which involves already a large space of possible muscle combinations (255) is in concert with that. To the best of our knowledge studies combining the possible sets of both sEMG feature selection and muscle selection are very rare and one which did cover both involves only 11 muscle combinations (Khan et al., 2021). In the present study, the muscle combination (i.e., MG + BF + GMax) used in sEMG feature selection process was chosen based on our previous study (Keleş and Yucesoy, 2020), which was shown to provide successful predictions, while minimizing the use of lower leg muscles. The feature combination IEMG+WL selected as a result, if put to the test with also other muscle combinations that stood out in Keleş and Yucesoy (2020) yields consistently the preferable results (see Supplementary Document 3 for details): for BF + MG + TA (the three muscle combination providing improved predictions without aiming at minimizing the use of lower leg muscles), for MG + TA (the successful combination with minimum muscle inputs) and for BF + MG + Gmax+RF + TA (the best-performing muscle combination), IEMG+WL shows the best performance among other feature combinations yielding strongly correlated predictions (minimally, *r*_position_ = 0.9161 and *r*_moment_ = 0.9739). Our comprehensive muscle selection analysis showed that 225 of 255 variations provide strong correlations for ankle position and moment prediction, ascribed to the flexibility of using all combinations of a wide range of muscles. A specific assessment seeking for the economic variation showed that PL alone provides the best performance in minimizing the number of muscle inputs. This is a lower leg muscle with limited accessibility but, strongly correlated predictions were shown to be achievable by utilizing solely upper leg muscles. The practical variation achieving the best performance by minimizing utilized upper leg muscles, is comprised of GMax+VM which are highly relevant for sEMG-based prosthesis controller development (Fleming et al., 2021). On the other hand, the flexible variation, i.e., MG + RF + VM showed that the best performance can be achieved by using a combination of upper and lower leg muscles.

The limitations of the present study need to be addressed. The position and moment predicting LSTMs were developed, trained and tested using data from healthy subjects only. Note that, the open database by Lencioni et al. (2019) involving nonnormalized sEMG amplitudes of various leg muscles is a rarity, but the lack of an open database that consists of amputee data limits further testing. However, Huang and Ferris (2012) reported that transtibial amputees are able to voluntarily activate their leg muscles with several of them producing activation profiles similar to healthy controls during voluntary dorsiflexion and plantar flexion. They also demonstrated that amputees are capable of generating consistent sEMG amplitude patterns from stride to stride, which supports the approach utilized in this study for developing position and moment predicting LSTMs. On the other hand, in case sEMG amplitude patterns show differences in amputees compared to those of healthy subjects, the proposed infrastructure can be adapted by re-training it with data collected from amputees. This will also facilitate a patient specific algorithm development. Also, the present study focuses on the prediction of ankle position and moment during level walking only, yet the LSTMs can be advanced for different locomotion tasks such as stair ascending and descending. Training their LSTM and using time-domain features extracted from nonnormalized sEMG amplitudes of eight muscles involving RF, BF, TA, and SO, Lu et al. (2022) achieved lower limb joint angles prediction for additional locomotion tasks. Therefore, the developed methodology is suitable for such prediction algorithms. Overall, the accuracy of ankle movement predictions should be studied after re-training the developed LSTM structure with a large database including nonnormalized sEMG amplitudes collected from lower extremity amputees and different locomotion tasks can be adapted. Note that unlike surface electrodes, electrodes within the socket of the prosthetic device may compromise user’s comfort, and long-term use may negatively affect sensor lifetime. The use of, e.g., sEMG knit band sensor (Lee et al., 2018) i.e., a silver-plated conductive yarn for electrodes knitted with a moisture-wicking technical yarn can make implementation much easier and improve comfort. Additionally, high density sEMG can help avoiding problems related to bipolar electrode placement (Ison et al., 2016) and improve data quality (Rojas-Martínez et al., 2012).

In bodily motion, humans have the sensation of position and moment changes in their limbs and such proprioception is essential to human motor control (Clites et al., 2018). Consequently, to support natural ambulation, powered prostheses require an advanced autonomous adaptation, which can be achieved with a real-time implementation of joint position and moment prediction (Fleming et al., 2021). The present study serves this purpose by utilizing nonnormalized sEMG amplitudes and LSTM architecture for ankle joint position and moment predictions in level-ground walking. This structure can be implemented in real-time robotic control applications (e.g., Li et al., 2021) and used to generate reference inputs for advanced powered prosthesis controllers such as impedance control (Aghasadeghi et al., 2013; Wu et al., 2022).

In conclusion, a novel LSTM approach utilizing exclusively lower limb nonnormalized sEMG amplitudes was developed. Its feasibility for predicting ankle angle and moment during level walking of a healthy population was evaluated by testing five time-domain features (IEMG, MAV, WAMP, RMS, and WL) and eight leg muscle combinations (TA, SO, MG, PL, RF, VM, BG, and GMax). PL and GMax+VM performed best in predicting ankle motion while minimizing the total number of sEMG inputs and minimizing the use of lower leg muscles, respectively. The best performing variation MG + RF + VM combines upper and lower leg muscle inputs. The versatile LSTM architecture utilizing nonnormalized sEMG for sensor inputs makes the algorithms get closer to being implemented in a real-time implementation. Moreover, the comprehensive testing protocol developed involving ranking of all muscle combinations according to their success will facilitate user specific solutions. However, in order to translate an offline application into online applicable powered ankle prosthesis control algorithms, the present implementation needs to be extended with other movement types, followed by training and testing of the algorithms for data collected from amputee participants in future studies.

## Data availability statement

The original contributions presented in the study are included in the article/Supplementary material, further inquiries can be directed to the corresponding author.

## Author contributions

AK, RT, and CY contributed to conception and design of the study. CY received the funding and edited sections of the manuscript. AK developed the algorithms, performed the analyses, and wrote the first draft of the manuscript. All authors contributed to manuscript revision, read, and approved the submitted version.

## Funding

This work was supported by the Scientific and Technological Research Council of Turkey (TÜBI TAK) under grant 120E272 to CY. Open access publication fees were covered by European University of Brain and Technology - Research and Innovation funded by the European Commision under grant 101035817.

## Conflict of interest

The authors declare that the research was conducted in the absence of any commercial or financial relationships that could be construed as a potential conflict of interest.

## Publisher’s note

All claims expressed in this article are solely those of the authors and do not necessarily represent those of their affiliated organizations, or those of the publisher, the editors and the reviewers. Any product that may be evaluated in this article, or claim that may be made by its manufacturer, is not guaranteed or endorsed by the publisher.
