# Supplementary material for: The use of nonnormalized surface EMG and feature inputs for LSTM-based powered ankle prosthesis control algorithm development
Source: Front Neurosci. 2023 Jul 3;17:1158280. doi: 10.3389/fnins.2023.1158280 (PMC10351874; doi:10.3389/fnins.2023.1158280)
Supplement: Supplementary file 1 [file Data_Sheet_1.DOCX]

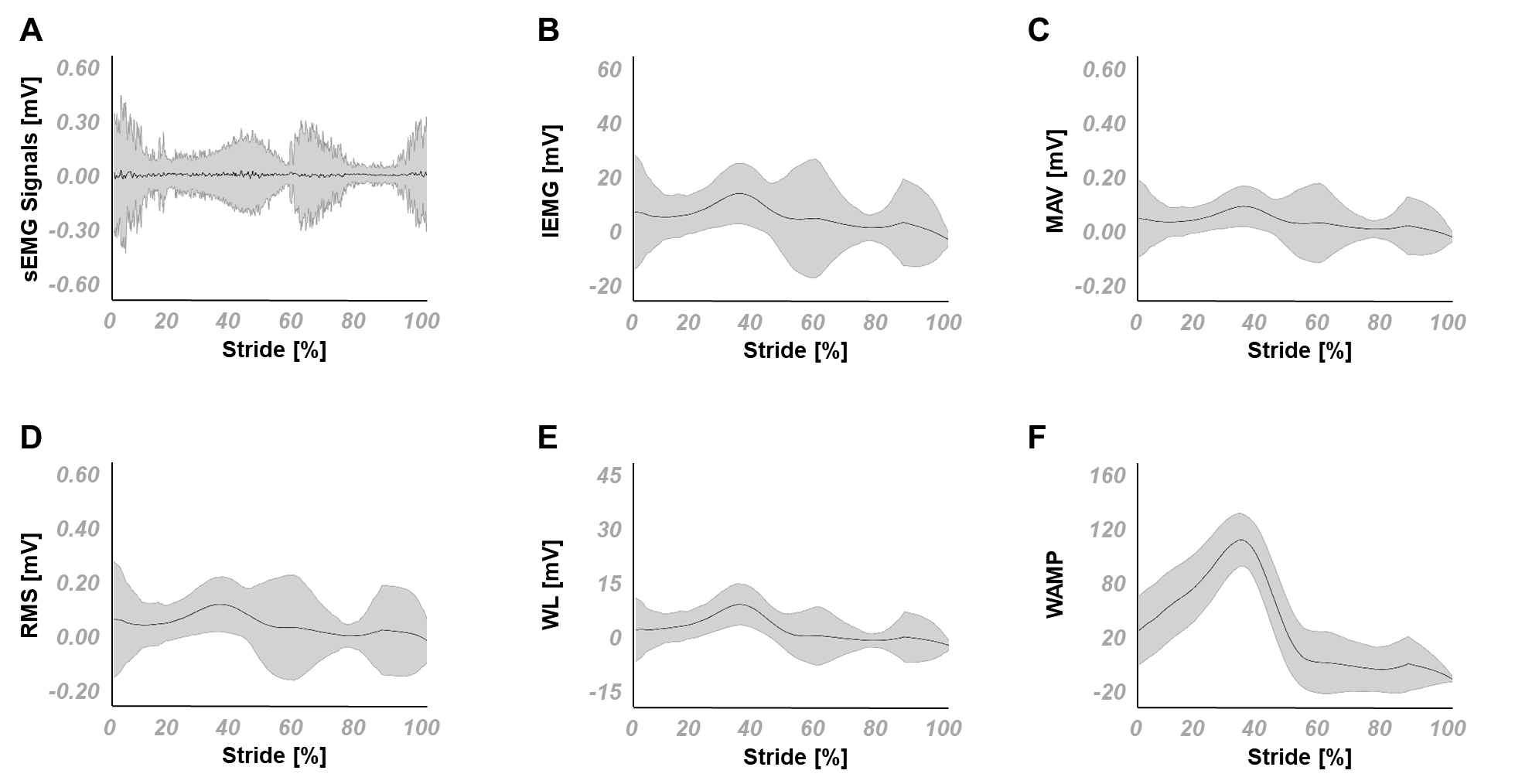


Fig. SD1-1. Mean and standard deviation of sEMG signals and its extracted features for the soleus, (A) the sEMG signals and the corresponding (B) IEMG, (C) MAV, (D) RMS, (E) WL and (F) WAMP features. IEMG: integrated EMG, MAV: mean absolute value, WAMP: Willison amplitude, RMS: root mean square; WL: waveform length.


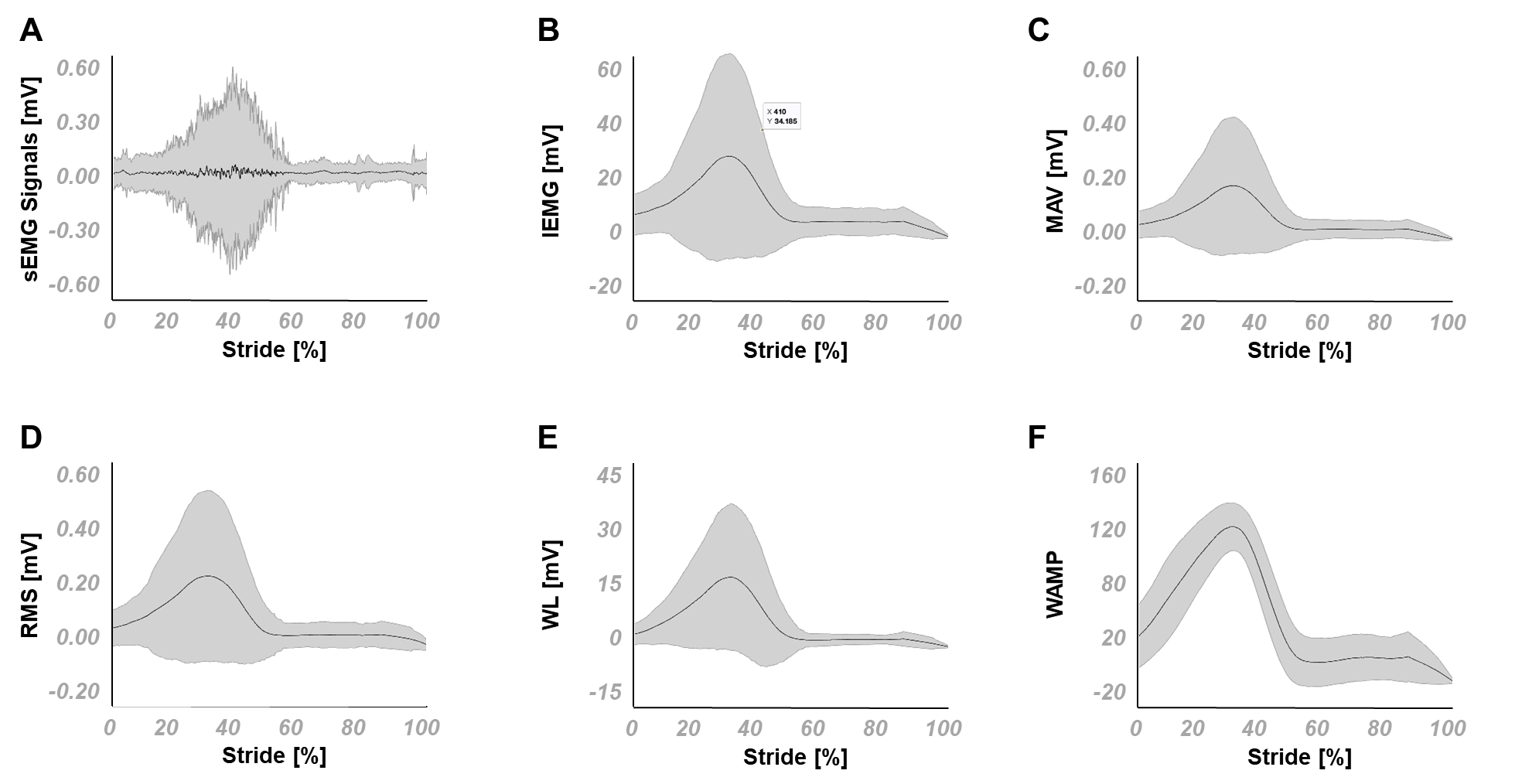


Fig. SD1-2. Mean and standard deviation of sEMG signals and its extracted features for the medial gastrocnemius, (A) the sEMG signals and the corresponding (B) IEMG, (C) MAV, (D) RMS, (E) WL and (F) WAMP features. IEMG: integrated EMG, MAV: mean absolute value, WAMP: Willison amplitude, RMS: root mean square; WL: waveform length*.*


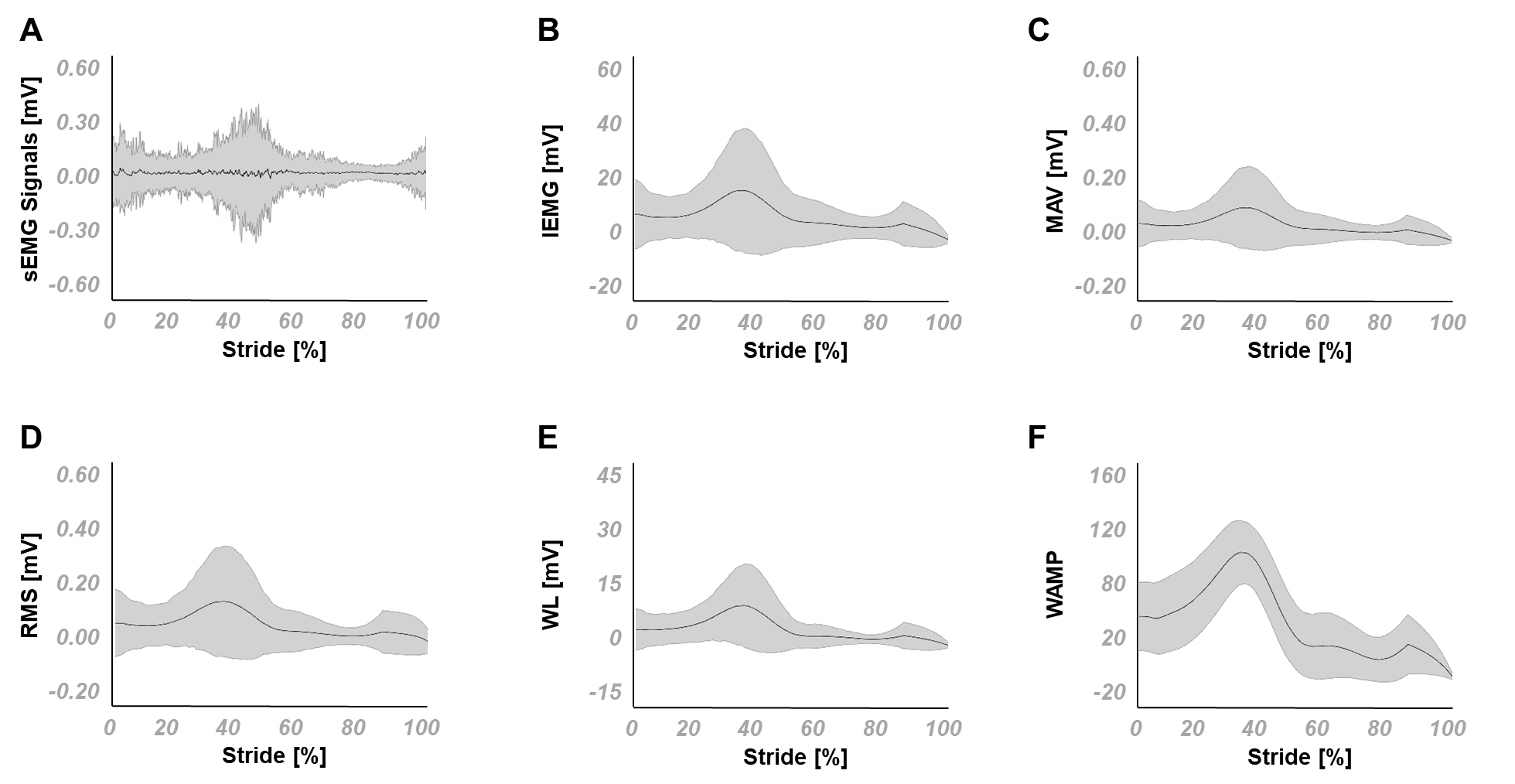


Fig. SD1-3. Mean and standard deviation of sEMG signals and its extracted features for the peroneus longus, (A) the sEMG signals and the corresponding (B) IEMG, (C) MAV, (D) RMS, (E) WL and (F) WAMP features. IEMG: integrated EMG, MAV: mean absolute value, WAMP: Willison amplitude, RMS: root mean square; WL: waveform length*.*


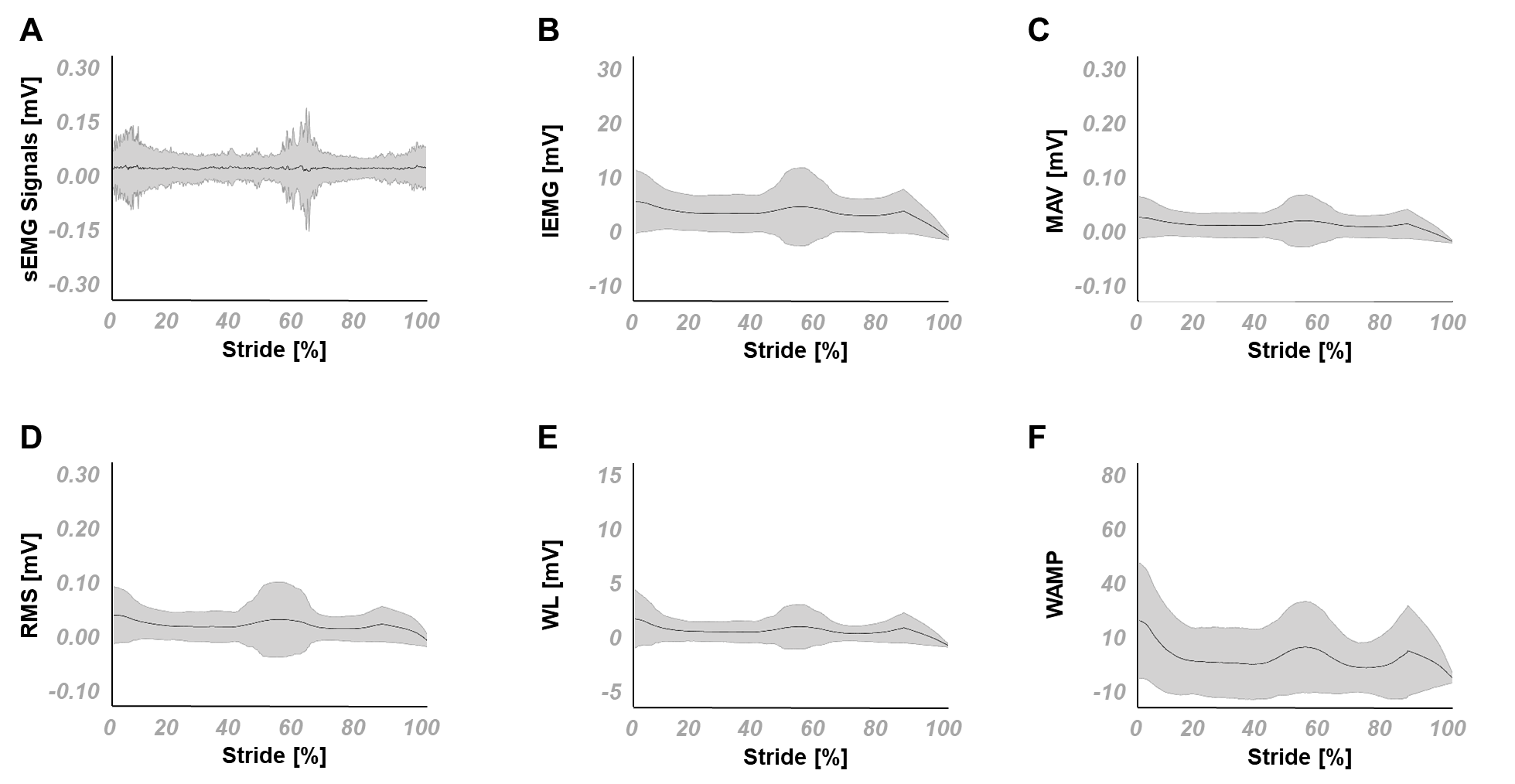


Fig. SD1-4. Mean and standard deviation of sEMG signals and its extracted features for the rectus femoris, (A) the sEMG signals and the corresponding (B) IEMG, (C) MAV, (D) RMS, (E) WL and (F) WAMP features. IEMG: integrated EMG, MAV: mean absolute value, WAMP: Willison amplitude, RMS: root mean square; WL: waveform length*.*


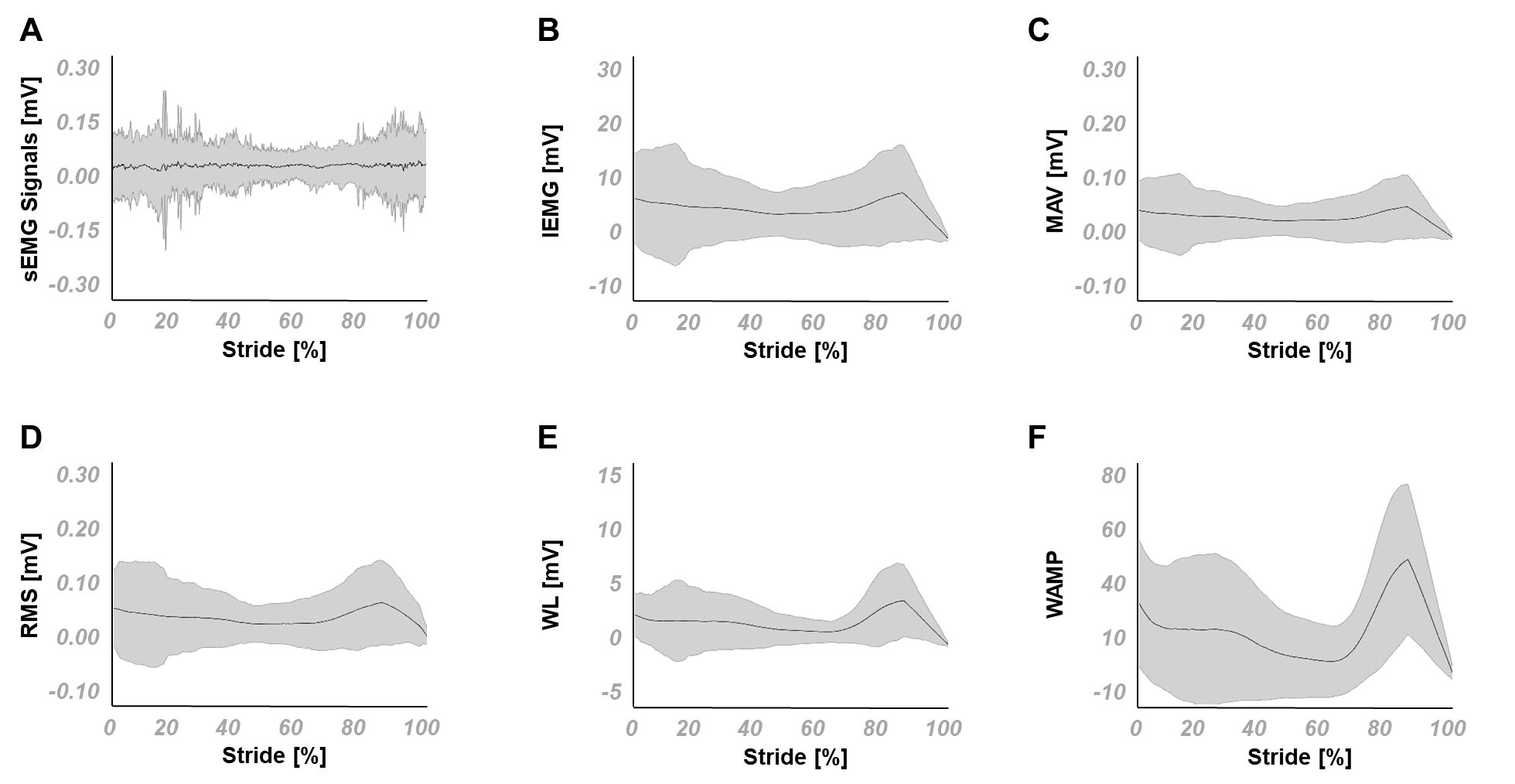


Fig. SD1-5. Mean and standard deviation of sEMG signals and its extracted features for the biceps femoris, (A) the sEMG signals and the corresponding (B) IEMG, (C) MAV, (D) RMS, (E) WL and (F) WAMP features. IEMG: integrated EMG, MAV: mean absolute value, WAMP: Willison amplitude, RMS: root mean square; WL: waveform length*.*


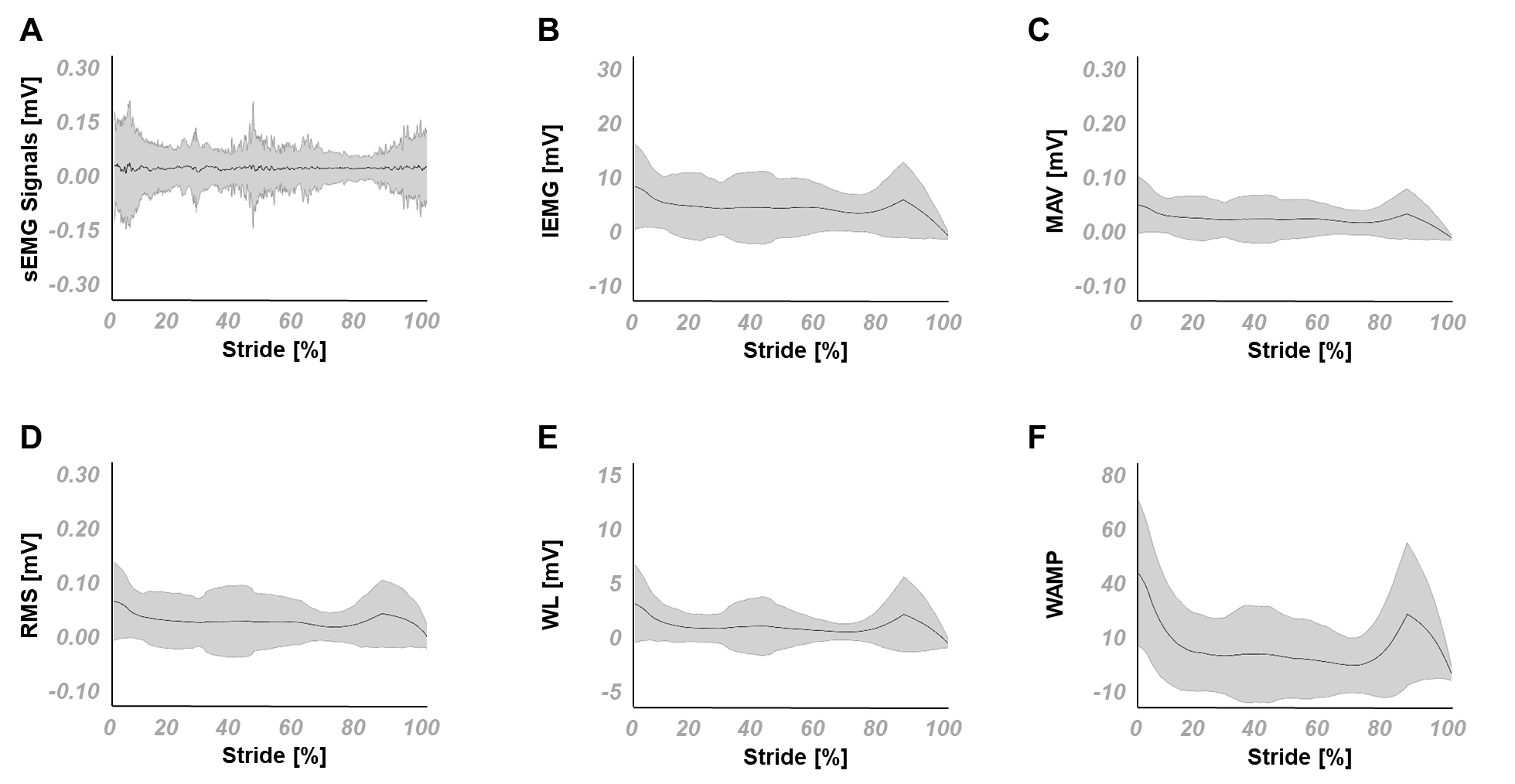


Fig. SD1-6. Mean and standard deviation of sEMG signals and its extracted features for the vastus medialis, (A) the sEMG signals and the corresponding (B) IEMG, (C) MAV, (D) RMS, (E) WL and (F) WAMP features. IEMG: integrated EMG, MAV: mean absolute value, WAMP: Willison amplitude, RMS: root mean square; WL: waveform length*.*


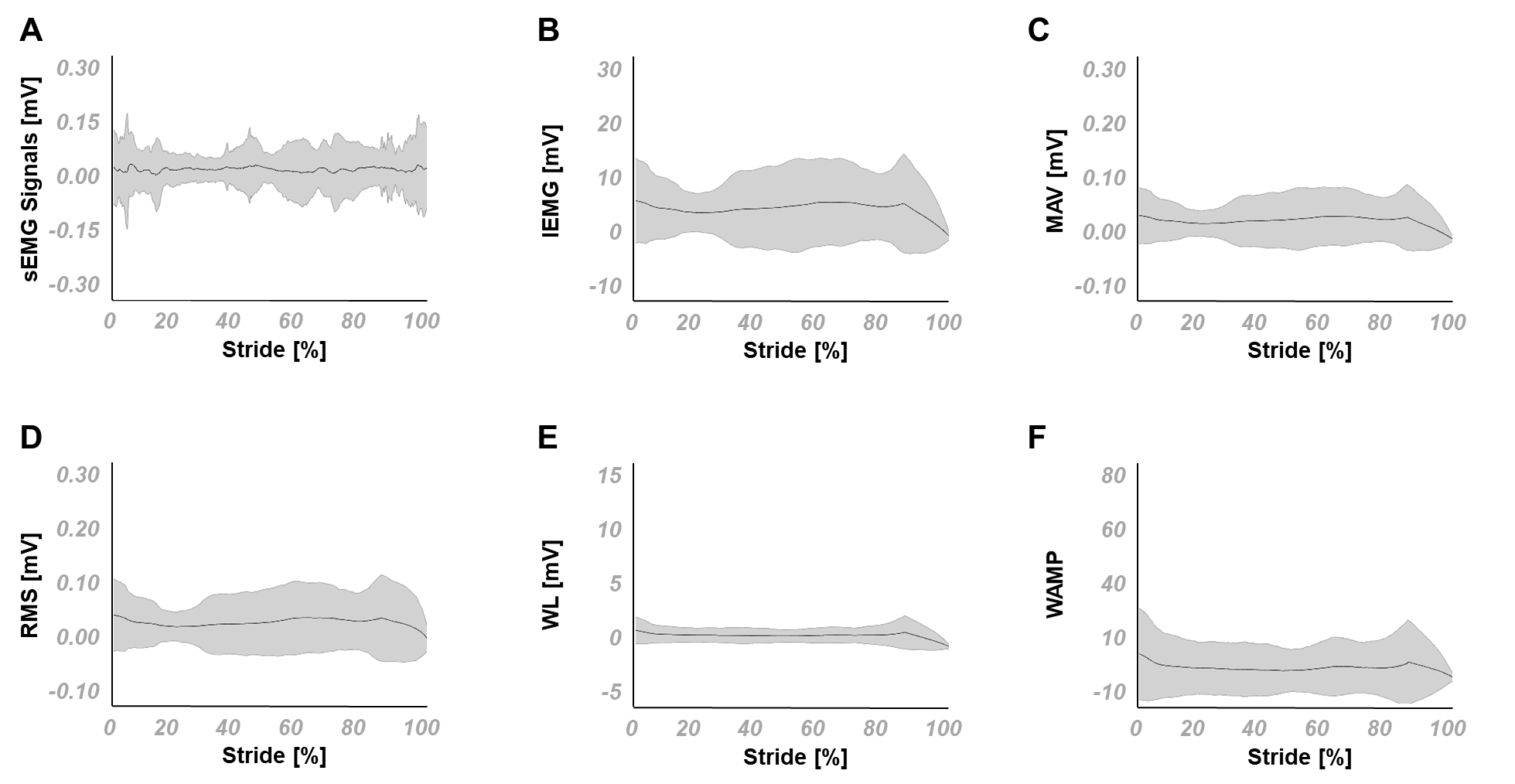


Fig. SD1-7. Mean and standard deviation of sEMG signals and its extracted features for the gluteus maximus, (A) the sEMG signals and the corresponding (B) IEMG, (C) MAV, (D) RMS, (E) WL and (F) WAMP features. IEMG: integrated EMG, MAV: mean absolute value, WAMP: Willison amplitude, RMS: root mean square; WL: waveform length*.*
