# Supplementary material for: The use of nonnormalized surface EMG and feature inputs for LSTM-based powered ankle prosthesis control algorithm development
Source: Front Neurosci. 2023 Jul 3;17:1158280. doi: 10.3389/fnins.2023.1158280 (PMC10351874; doi:10.3389/fnins.2023.1158280)
Supplement: Supplementary file 2 [file Data_Sheet_2.DOCX]

**Table SD2-1.** The comparison of the muscle variations. Grey shaded cells show the muscle variations that do not provide strongly correlated position and moment predictions (i.e., r < 0.90). Yellow shaded cells show the only upper leg muscle variations that provide strongly correlated position and moment predictions (i.e., r >= 0.90). TA: tibialis anterior, SO: soleus, MG: medial gastrocnemius, PL: peroneus longus, RF: rectus femoris, BF: biceps femoris, VM: vastus medialis GMax: gluteus maximus, RMSE: root-mean-square error.

| **Rank** | **Muscle Variations** | **Position Correlation  [r]** | **Moment Correlation  [r]** | **Position RMSE  [deg]** | **Moment RMSE  [Nm/kg]** | **Position SPM [%GC]** | **Moment SPM [%GC]** | **Miscorrelation Score** | **Error  Score** | **SPM Score** | **Overall Error Score** |
| --- | --- | --- | --- | --- | --- | --- | --- | --- | --- | --- | --- |
| 1 | MG+RF+VM | 0.9099±0.0711 | 0.9707±0.0784 | 4.5923±1.4815 | 0.1072±0.0660 | 0.0300 | 0.0400 | 0.0597 | 0.8819 | 0.0350 | 0.0018 |
| 2 | GMax+RF+SO | 0.8978±0.0884 | 0.9716±0.0400 | 4.8759±1.6262 | 0.1151±0.0515 | 0.0500 | 0.0100 | 0.0653 | 0.9415 | 0.0300 | 0.0018 |
| 3 | BF+MG+GMax+PL+RF+SO | 0.9134±0.0938 | 0.9714±0.0600 | 4.3291±1.6537 | 0.1067±0.0624 | 0.0700 | 0.0200 | 0.0576 | 0.8543 | 0.0450 | 0.0022 |
| 4 | BF+MG+GMax+RF+SO+TA+VM | 0.9246±0.0614 | 0.9803±0.0270 | 4.1877±1.5685 | 0.1012±0.0514 | 0.1200 | 0.0000 | 0.0475 | 0.8182 | 0.0600 | 0.0023 |
| 5 | MG+GMax+PL+VM | 0.9101±0.0793 | 0.9725±0.0753 | 4.5901±1.6014 | 0.1085±0.0601 | 0.1000 | 0.0000 | 0.0587 | 0.8869 | 0.0500 | 0.0026 |
| 6 | BF+MG+GMax | 0.9006±0.0831 | 0.9742±0.0503 | 4.6781±1.7030 | 0.1094±0.0610 | 0.1000 | 0.0000 | 0.0626 | 0.8991 | 0.0500 | 0.0028 |
| 7 | BF+MG+GMax+PL+RF+SO+TA+VM | 0.9205±0.0788 | 0.9695±0.0862 | 4.2841±1.6202 | 0.1060±0.0669 | 0.0100 | 0.1200 | 0.0550 | 0.8471 | 0.0650 | 0.0030 |
| 8 | MG+GMax+RF+TA | 0.9123±0.0730 | 0.9789±0.0331 | 4.5078±1.6104 | 0.1034±0.0551 | 0.1100 | 0.0300 | 0.0544 | 0.8582 | 0.0700 | 0.0033 |
| 9 | MG+GMax+PL+RF+SO+TA | 0.9267±0.0548 | 0.9733±0.0490 | 4.1133±1.4718 | 0.1079±0.0573 | 0.0500 | 0.1100 | 0.0500 | 0.8384 | 0.0800 | 0.0034 |
| 10 | MG+GMax+TA | 0.9164±0.0670 | 0.9782±0.0276 | 4.4843±1.5643 | 0.1059±0.0538 | 0.1200 | 0.0300 | 0.0527 | 0.8661 | 0.0750 | 0.0034 |
| 11 | RF+TA | 0.9174±0.0689 | 0.9776±0.0238 | 4.6396±1.5971 | 0.1081±0.0439 | 0.0300 | 0.1200 | 0.0525 | 0.8901 | 0.0750 | 0.0035 |
| 12 | MG+SO+TA | 0.9197±0.0587 | 0.9769±0.0373 | 4.4188±1.6482 | 0.1072±0.0529 | 0.1100 | 0.0500 | 0.0517 | 0.8651 | 0.0800 | 0.0036 |
| 13 | BF+MG+GMax+PL+SO+TA | 0.9166±0.0938 | 0.9760±0.0529 | 4.3324±1.6361 | 0.1070±0.0595 | 0.0200 | 0.1400 | 0.0537 | 0.8559 | 0.0800 | 0.0037 |
| 14 | BF+PL+RF+TA | 0.9161±0.0757 | 0.9734±0.0364 | 4.4649±1.5828 | 0.1119±0.0496 | 0.1000 | 0.0500 | 0.0552 | 0.8887 | 0.0750 | 0.0037 |
| 15 | PL+SO+TA+VM | 0.9216±0.0615 | 0.9768±0.0291 | 4.4417±1.4206 | 0.1104±0.0500 | 0.1200 | 0.0600 | 0.0508 | 0.8803 | 0.0900 | 0.0040 |
| 16 | MG+PL+TA | 0.9203±0.0732 | 0.9757±0.0498 | 4.3252±1.5455 | 0.1083±0.0599 | 0.1200 | 0.0700 | 0.0520 | 0.8605 | 0.0950 | 0.0043 |
| 17 | MG+GMax+PL+RF+TA | 0.9099±0.1285 | 0.9779±0.0290 | 4.3088±1.6608 | 0.1068±0.0500 | 0.1200 | 0.0600 | 0.0561 | 0.8528 | 0.0900 | 0.0043 |
| 18 | BF+MG+RF+SO+TA+VM | 0.9169±0.0831 | 0.9754±0.0553 | 4.2226±1.6019 | 0.1048±0.0536 | 0.1400 | 0.0600 | 0.0539 | 0.8363 | 0.1000 | 0.0045 |
| 19 | MG+GMax+PL+RF+SO+VM | 0.9136±0.0699 | 0.9734±0.0476 | 4.5057±1.4465 | 0.1078±0.0570 | 0.0900 | 0.1000 | 0.0565 | 0.8759 | 0.0950 | 0.0047 |
| 20 | MG+GMax+RF+SO+TA | 0.9196±0.0735 | 0.9725±0.0524 | 4.3227±1.5550 | 0.1090±0.0558 | 0.0600 | 0.1500 | 0.0539 | 0.8631 | 0.1050 | 0.0049 |
| 21 | BF+GMax+VM | 0.8904±0.1091 | 0.9668±0.0646 | 5.0332±1.7615 | 0.1212±0.0652 | 0.1300 | 0.0100 | 0.0714 | 0.9817 | 0.0700 | 0.0049 |
| 22 | GMax+PL+RF | 0.9068±0.0759 | 0.9689±0.0859 | 4.7873±1.6091 | 0.1143±0.0567 | 0.1700 | 0.0000 | 0.0622 | 0.9297 | 0.0850 | 0.0049 |
| 23 | BF+MG+PL+RF | 0.9102±0.1012 | 0.9692±0.1117 | 4.5424±1.6870 | 0.1074±0.0657 | 0.0800 | 0.1100 | 0.0603 | 0.8778 | 0.0950 | 0.0050 |
| 24 | BF+MG+GMax+PL+VM | 0.9097±0.0738 | 0.9788±0.0280 | 4.4883±1.4887 | 0.1065±0.0479 | 0.1000 | 0.1100 | 0.0558 | 0.8689 | 0.1050 | 0.0051 |
| 25 | MG+GMax+RF+SO+VM | 0.9119±0.0676 | 0.9765±0.0326 | 4.5431±1.6573 | 0.1079±0.0512 | 0.1000 | 0.1100 | 0.0558 | 0.8799 | 0.1050 | 0.0052 |
| 26 | BF+RF+TA | 0.9164±0.0675 | 0.9744±0.0406 | 4.5841±1.5756 | 0.1124±0.0485 | 0.0600 | 0.1500 | 0.0546 | 0.9023 | 0.1050 | 0.0052 |
| 27 | BF+PL+SO+VM | 0.9156±0.0641 | 0.9649±0.0927 | 4.5507±1.4057 | 0.1183±0.0742 | 0.0400 | 0.1600 | 0.0597 | 0.9231 | 0.1000 | 0.0055 |
| 28 | MG+PL+RF+SO+TA | 0.9212±0.0628 | 0.9589±0.1556 | 4.3659±1.4639 | 0.1113±0.0748 | 0.1600 | 0.0500 | 0.0600 | 0.8767 | 0.1050 | 0.0055 |
| 29 | BF+MG+GMax+PL+SO+VM | 0.9213±0.0737 | 0.9748±0.0422 | 4.2727±1.5538 | 0.1077±0.0564 | 0.0100 | 0.2400 | 0.0520 | 0.8530 | 0.1250 | 0.0055 |
| 30 | BF+MG+VM | 0.9201±0.0675 | 0.9769±0.0371 | 4.4867±1.6031 | 0.1083±0.0548 | 0.0300 | 0.2300 | 0.0515 | 0.8761 | 0.1300 | 0.0059 |
| 31 | MG+PL+RF+SO+TA+VM | 0.9243±0.0581 | 0.9766±0.0408 | 4.3333±1.4006 | 0.1037±0.0562 | 0.2000 | 0.0900 | 0.0495 | 0.8425 | 0.1450 | 0.0061 |
| 32 | MG+GMax+SO+TA | 0.9179±0.0738 | 0.9709±0.0752 | 4.4712±1.6520 | 0.1081±0.0668 | 0.2000 | 0.0500 | 0.0556 | 0.8738 | 0.1250 | 0.0061 |
| 33 | BF+MG+GMax+RF+VM | 0.9113±0.1388 | 0.9638±0.1056 | 4.4414±1.8052 | 0.1131±0.0762 | 0.0200 | 0.2000 | 0.0624 | 0.8913 | 0.1100 | 0.0061 |
| 34 | BF+MG+RF+TA+VM | 0.9230±0.0590 | 0.9780±0.0295 | 4.4489±1.4854 | 0.1038±0.0483 | 0.2900 | 0.0000 | 0.0495 | 0.8541 | 0.1450 | 0.0061 |
| 35 | MG+GMax+PL+RF | 0.9087±0.0804 | 0.9723±0.0393 | 4.5340±1.5885 | 0.1130±0.0615 | 0.1500 | 0.0800 | 0.0595 | 0.8999 | 0.1150 | 0.0062 |
| 36 | MG+GMax+PL+TA | 0.9182±0.0634 | 0.9721±0.0543 | 4.3893±1.4984 | 0.1106±0.0625 | 0.1600 | 0.1000 | 0.0548 | 0.8761 | 0.1300 | 0.0062 |
| 37 | MG+PL+SO | 0.9181±0.0649 | 0.9691±0.0839 | 4.4428±1.4805 | 0.1119±0.0637 | 0.0600 | 0.1900 | 0.0564 | 0.8866 | 0.1250 | 0.0063 |
| 38 | BF+RF+SO+TA+VM | 0.9222±0.0623 | 0.9610±0.1186 | 4.3213±1.4126 | 0.1164±0.0778 | 0.1500 | 0.0900 | 0.0584 | 0.8932 | 0.1200 | 0.0063 |
| 39 | BF+MG+SO+TA | 0.9242±0.0641 | 0.9774±0.0375 | 4.2392±1.5807 | 0.1074±0.0527 | 0.0200 | 0.2800 | 0.0492 | 0.8485 | 0.1500 | 0.0063 |
| 40 | BF+MG+GMax+TA+VM | 0.9196±0.0606 | 0.9714±0.0593 | 4.4385±1.4616 | 0.1091±0.0564 | 0.0300 | 0.2400 | 0.0545 | 0.8747 | 0.1350 | 0.0064 |
| 41 | SO+TA+VM | 0.9240±0.0537 | 0.9774±0.0276 | 4.4253±1.3715 | 0.1081±0.0476 | 0.0100 | 0.3000 | 0.0493 | 0.8694 | 0.1550 | 0.0066 |
| 42 | BF+MG+PL+TA+VM | 0.9282±0.0595 | 0.9751±0.0551 | 4.2314±1.3883 | 0.1064±0.0543 | 0.1400 | 0.1900 | 0.0484 | 0.8437 | 0.1650 | 0.0067 |
| 43 | GMax+SO+VM | 0.9036±0.0739 | 0.9719±0.0524 | 4.8336±1.5722 | 0.1130±0.0521 | 0.0500 | 0.1900 | 0.0623 | 0.9289 | 0.1200 | 0.0069 |
| 44 | BF+GMax+SO+VM | 0.9082±0.0676 | 0.9735±0.0287 | 4.6375±1.6045 | 0.1145±0.0515 | 0.1900 | 0.0700 | 0.0591 | 0.9160 | 0.1300 | 0.0070 |
| 45 | SO+VM | 0.9102±0.0664 | 0.9757±0.0292 | 4.7049±1.4715 | 0.1126±0.0501 | 0.0500 | 0.2300 | 0.0570 | 0.9148 | 0.1400 | 0.0073 |
| 46 | BF+MG+PL+RF+VM | 0.9055±0.0933 | 0.9714±0.0792 | 4.5869±1.7207 | 0.1076±0.0611 | 0.1900 | 0.0800 | 0.0615 | 0.8830 | 0.1350 | 0.0073 |
| 47 | MG+PL+SO+TA+VM | 0.9261±0.0737 | 0.9749±0.0454 | 4.1641±1.5317 | 0.1090±0.0529 | 0.0500 | 0.3000 | 0.0495 | 0.8478 | 0.1750 | 0.0073 |
| 48 | BF+MG+PL+RF+SO | 0.9011±0.1679 | 0.9738±0.0583 | 4.4659±1.8788 | 0.1073±0.0579 | 0.1400 | 0.1300 | 0.0626 | 0.8700 | 0.1350 | 0.0073 |
| 49 | BF+MG+RF+VM | 0.9133±0.0816 | 0.9784±0.0403 | 4.4769±1.6615 | 0.1036±0.0513 | 0.0600 | 0.2600 | 0.0542 | 0.8560 | 0.1600 | 0.0074 |
| 50 | BF+GMax+SO | 0.9162±0.0658 | 0.9688±0.0624 | 4.5551±1.6202 | 0.1186±0.0578 | 0.0300 | 0.2500 | 0.0575 | 0.9248 | 0.1400 | 0.0074 |
| 51 | BF+MG+RF+SO+TA | 0.9200±0.0693 | 0.9801±0.0358 | 4.3880±1.6149 | 0.1006±0.0498 | 0.2000 | 0.1600 | 0.0500 | 0.8351 | 0.1800 | 0.0075 |
| 52 | GMax+RF+TA | 0.9069±0.0892 | 0.9725±0.0414 | 4.7159±1.7024 | 0.1144±0.0499 | 0.0300 | 0.2400 | 0.0603 | 0.9232 | 0.1350 | 0.0075 |
| 53 | GMax+PL+SO+TA+VM | 0.9192±0.0582 | 0.9783±0.0250 | 4.4630±1.4955 | 0.1067±0.0487 | 0.0500 | 0.2900 | 0.0513 | 0.8673 | 0.1700 | 0.0076 |
| 54 | BF+MG+GMax+RF+SO+VM | 0.9095±0.0842 | 0.9772±0.0344 | 4.5465±1.7961 | 0.1058±0.0533 | 0.1900 | 0.1200 | 0.0567 | 0.8717 | 0.1550 | 0.0077 |
| 55 | MG+GMax | 0.8951±0.0931 | 0.9762±0.0380 | 4.8409±1.7516 | 0.1098±0.0531 | 0.0300 | 0.2300 | 0.0644 | 0.9165 | 0.1300 | 0.0077 |
| 56 | MG+PL+RF+VM | 0.9116±0.0702 | 0.9798±0.0296 | 4.5032±1.4432 | 0.1040±0.0498 | 0.1300 | 0.2000 | 0.0543 | 0.8602 | 0.1650 | 0.0077 |
| 57 | BF+MG+PL+SO+VM | 0.9190±0.0719 | 0.9709±0.0699 | 4.4087±1.4642 | 0.1104±0.0628 | 0.1800 | 0.1400 | 0.0550 | 0.8772 | 0.1600 | 0.0077 |
| 58 | GMax+RF | 0.8916±0.0832 | 0.9687±0.0578 | 5.0415±1.7077 | 0.1175±0.0534 | 0.1200 | 0.1100 | 0.0698 | 0.9674 | 0.1150 | 0.0078 |
| 59 | MG+GMax+PL+TA+VM | 0.9095±0.0859 | 0.9764±0.0585 | 4.4972±1.6279 | 0.1043±0.0604 | 0.2000 | 0.1200 | 0.0570 | 0.8608 | 0.1600 | 0.0079 |
| 60 | MG+GMax+SO+VM | 0.9138±0.0666 | 0.9754±0.0356 | 4.4813±1.5270 | 0.1124±0.0573 | 0.1100 | 0.2100 | 0.0554 | 0.8923 | 0.1600 | 0.0079 |
| 61 | GMax+PL+VM | 0.9069±0.0919 | 0.9706±0.0781 | 4.7042±1.5726 | 0.1152±0.0672 | 0.1600 | 0.1200 | 0.0613 | 0.9253 | 0.1400 | 0.0079 |
| 62 | BF+MG+GMax+RF+SO | 0.9057±0.0805 | 0.9704±0.0852 | 4.5828±1.7318 | 0.1088±0.0659 | 0.2600 | 0.0300 | 0.0619 | 0.8875 | 0.1450 | 0.0080 |
| 63 | BF+MG+PL+RF+SO+TA | 0.9232±0.0655 | 0.9763±0.0476 | 4.3223±1.4507 | 0.1053±0.0585 | 0.1000 | 0.2800 | 0.0502 | 0.8480 | 0.1900 | 0.0081 |
| 64 | PL+TA+VM | 0.9148±0.0636 | 0.9753±0.0267 | 4.5563±1.4274 | 0.1138±0.0501 | 0.0700 | 0.2600 | 0.0549 | 0.9053 | 0.1650 | 0.0082 |
| 65 | BF+GMax+TA | 0.9134±0.0685 | 0.9707±0.0405 | 4.6710±1.5956 | 0.1160±0.0564 | 0.0700 | 0.2400 | 0.0579 | 0.9254 | 0.1550 | 0.0083 |
| 66 | RF+SO+TA | 0.9158±0.0784 | 0.9678±0.0802 | 4.5163±1.7862 | 0.1138±0.0649 | 0.1600 | 0.1700 | 0.0582 | 0.9014 | 0.1650 | 0.0087 |
| 67 | GMax+RF+TA+VM | 0.9223±0.0571 | 0.9627±0.1167 | 4.4178±1.3645 | 0.1196±0.0707 | 0.0200 | 0.3100 | 0.0575 | 0.9156 | 0.1650 | 0.0087 |
| 68 | MG+GMax+RF | 0.8994±0.0854 | 0.9768±0.0309 | 4.8072±1.6391 | 0.1086±0.0479 | 0.1400 | 0.1700 | 0.0619 | 0.9084 | 0.1550 | 0.0087 |
| 69 | PL | 0.9001±0.0729 | 0.9703±0.0657 | 4.9396±1.6826 | 0.1190±0.0563 | 0.1100 | 0.1700 | 0.0649 | 0.9636 | 0.1400 | 0.0087 |
| 70 | GMax+SO | 0.8879±0.0989 | 0.9708±0.0482 | 4.9708±1.7860 | 0.1177±0.0580 | 0.0900 | 0.1700 | 0.0707 | 0.9613 | 0.1300 | 0.0088 |
| 71 | BF+MG+TA | 0.9199±0.0682 | 0.9787±0.0306 | 4.4577±1.5804 | 0.1028±0.0494 | 0.1400 | 0.2700 | 0.0507 | 0.8509 | 0.2050 | 0.0088 |
| 72 | BF+PL+TA+VM | 0.9162±0.0727 | 0.9727±0.0324 | 4.5415±1.4349 | 0.1166±0.0595 | 0.1800 | 0.1700 | 0.0556 | 0.9153 | 0.1750 | 0.0089 |
| 73 | BF+MG+GMax+RF+TA | 0.9161±0.0792 | 0.9739±0.0445 | 4.2673±1.6024 | 0.1080±0.0547 | 0.0200 | 0.3600 | 0.0550 | 0.8537 | 0.1900 | 0.0089 |
| 74 | GMax | 0.8792±0.1092 | 0.9732±0.0259 | 5.1680±1.9614 | 0.1161±0.0450 | 0.0800 | 0.1700 | 0.0738 | 0.9739 | 0.1250 | 0.0090 |
| 75 | GMax+PL+RF+SO+VM | 0.9104±0.0756 | 0.9703±0.0550 | 4.6538±1.6926 | 0.1145±0.0625 | 0.2600 | 0.0700 | 0.0597 | 0.9176 | 0.1650 | 0.0090 |
| 76 | MG+RF+TA | 0.9202±0.0619 | 0.9806±0.0294 | 4.4075±1.6188 | 0.0999±0.0482 | 0.1900 | 0.2500 | 0.0496 | 0.8342 | 0.2200 | 0.0091 |
| 77 | MG+RF+SO+TA+VM | 0.9078±0.0878 | 0.9772±0.0404 | 4.5132±1.6826 | 0.1048±0.0546 | 0.2900 | 0.0800 | 0.0575 | 0.8644 | 0.1850 | 0.0092 |
| 78 | MG+RF+SO+TA | 0.9144±0.0672 | 0.9775±0.0407 | 4.4121±1.5927 | 0.1041±0.0526 | 0.2500 | 0.1500 | 0.0540 | 0.8518 | 0.2000 | 0.0092 |
| 79 | BF+MG+GMax+PL+TA | 0.9117±0.1233 | 0.9722±0.0457 | 4.4084±1.8329 | 0.1116±0.0616 | 0.0300 | 0.3300 | 0.0581 | 0.8820 | 0.1800 | 0.0092 |
| 80 | GMax+PL+RF+VM | 0.9084±0.0690 | 0.9718±0.0422 | 4.7488±1.4566 | 0.1165±0.0571 | 0.2100 | 0.1200 | 0.0599 | 0.9350 | 0.1650 | 0.0092 |
| 81 | BF+MG+PL+VM | 0.9115±0.0896 | 0.9748±0.0668 | 4.5681±1.5291 | 0.1072±0.0569 | 0.3400 | 0.0300 | 0.0569 | 0.8795 | 0.1850 | 0.0093 |
| 82 | MG+TA+VM | 0.9235±0.0554 | 0.9813±0.0247 | 4.3535±1.3910 | 0.1003±0.0452 | 0.1900 | 0.2800 | 0.0476 | 0.8306 | 0.2350 | 0.0093 |
| 83 | BF+MG+GMax+PL+SO+TA+VM | 0.9106±0.1072 | 0.9759±0.0363 | 4.3949±1.6559 | 0.1070±0.0516 | 0.2300 | 0.1500 | 0.0568 | 0.8619 | 0.1900 | 0.0093 |
| 84 | RF+SO+TA+VM | 0.9237±0.0555 | 0.9720±0.0502 | 4.4245±1.6611 | 0.1136±0.0575 | 0.2500 | 0.1500 | 0.0522 | 0.8917 | 0.2000 | 0.0093 |
| 85 | SO | 0.9002±0.0803 | 0.9756±0.0244 | 4.8927±1.7017 | 0.1144±0.0446 | 0.1300 | 0.1900 | 0.0621 | 0.9403 | 0.1600 | 0.0093 |
| 86 | BF+RF+SO+TA | 0.9130±0.0691 | 0.9753±0.0380 | 4.6492±1.8454 | 0.1119±0.0480 | 0.3100 | 0.0600 | 0.0558 | 0.9065 | 0.1850 | 0.0094 |
| 87 | BF+TA+VM | 0.9194±0.0552 | 0.9694±0.0705 | 4.5901±1.5236 | 0.1157±0.0585 | 0.0900 | 0.2800 | 0.0556 | 0.9163 | 0.1850 | 0.0094 |
| 88 | MG+RF | 0.9081±0.0721 | 0.9793±0.0255 | 4.7208±1.5978 | 0.1041±0.0455 | 0.2600 | 0.1200 | 0.0563 | 0.8816 | 0.1900 | 0.0094 |
| 89 | MG+GMax+RF+SO | 0.9066±0.0746 | 0.9756±0.0357 | 4.7051±1.7364 | 0.1076±0.0543 | 0.2800 | 0.0800 | 0.0589 | 0.8944 | 0.1800 | 0.0095 |
| 90 | BF+GMax+RF | 0.8953±0.0794 | 0.9686±0.0546 | 5.0077±1.7018 | 0.1187±0.0601 | 0.1300 | 0.1600 | 0.0681 | 0.9690 | 0.1450 | 0.0096 |
| 91 | MG+GMax+PL+SO+VM | 0.9177±0.0669 | 0.9645±0.1168 | 4.3902±1.4979 | 0.1157±0.0791 | 0.0400 | 0.3300 | 0.0589 | 0.8970 | 0.1850 | 0.0098 |
| 92 | BF+MG+SO+VM | 0.9114±0.0785 | 0.9807±0.0259 | 4.5606±1.6139 | 0.1034±0.0495 | 0.1500 | 0.2700 | 0.0539 | 0.8633 | 0.2100 | 0.0098 |
| 93 | MG+PL+RF+TA+VM | 0.9172±0.0676 | 0.9766±0.0349 | 4.4166±1.5188 | 0.1068±0.0503 | 0.0800 | 0.3500 | 0.0531 | 0.8632 | 0.2150 | 0.0099 |
| 94 | BF+GMax+PL+RF+TA+VM | 0.9195±0.0631 | 0.9706±0.0663 | 4.4626±1.4859 | 0.1112±0.0577 | 0.4100 | 0.0000 | 0.0549 | 0.8856 | 0.2050 | 0.0100 |
| 95 | PL+RF+SO+TA+VM | 0.9269±0.0590 | 0.9759±0.0355 | 4.2195±1.5076 | 0.1112±0.0498 | 0.2000 | 0.2800 | 0.0486 | 0.8621 | 0.2400 | 0.0101 |
| 96 | BF+PL+RF+VM | 0.9114±0.0803 | 0.9704±0.0551 | 4.5794±1.5454 | 0.1170±0.0583 | 0.0600 | 0.3100 | 0.0591 | 0.9206 | 0.1850 | 0.0101 |
| 97 | GMax+PL+RF+TA | 0.9147±0.0698 | 0.9690±0.0596 | 4.5329±1.5407 | 0.1167±0.0595 | 0.0300 | 0.3500 | 0.0582 | 0.9149 | 0.1900 | 0.0101 |
| 98 | BF+MG+GMax+SO | 0.8995±0.1596 | 0.9778±0.0284 | 4.5888±1.9496 | 0.1099±0.0483 | 0.0600 | 0.3100 | 0.0613 | 0.8925 | 0.1850 | 0.0101 |
| 99 | MG+PL | 0.9119±0.0708 | 0.9800±0.0239 | 4.6220±1.5841 | 0.1050±0.0460 | 0.1900 | 0.2400 | 0.0540 | 0.8757 | 0.2150 | 0.0102 |
| 100 | BF+MG+SO+TA+VM | 0.9161±0.0754 | 0.9789±0.0406 | 4.3361±1.6940 | 0.1026±0.0500 | 0.2800 | 0.1900 | 0.0525 | 0.8383 | 0.2350 | 0.0103 |
| 101 | PL+VM | 0.8993±0.1182 | 0.9765±0.0297 | 4.8307±1.6342 | 0.1135±0.0497 | 0.1000 | 0.2600 | 0.0621 | 0.9306 | 0.1800 | 0.0104 |
| 102 | GMax+SO+TA | 0.9194±0.0735 | 0.9659±0.0906 | 4.4673±1.4984 | 0.1168±0.0640 | 0.0600 | 0.3400 | 0.0574 | 0.9089 | 0.2000 | 0.0104 |
| 103 | BF+GMax+PL+RF+TA | 0.9230±0.0700 | 0.9718±0.0740 | 4.3901±1.5002 | 0.1091±0.0543 | 0.3300 | 0.1300 | 0.0526 | 0.8700 | 0.2300 | 0.0105 |
| 104 | GMax+PL+RF+SO+TA+VM | 0.9284±0.0470 | 0.9716±0.0719 | 4.2607±1.4340 | 0.1144±0.0598 | 0.0600 | 0.4200 | 0.0500 | 0.8792 | 0.2400 | 0.0105 |
| 105 | BF+TA | 0.9107±0.1007 | 0.9771±0.0271 | 4.6781±1.7578 | 0.1096±0.0458 | 0.0800 | 0.3400 | 0.0561 | 0.8999 | 0.2100 | 0.0106 |
| 106 | GMax+VM | 0.9010±0.0805 | 0.9718±0.0326 | 4.8927±1.6140 | 0.1193±0.0501 | 0.0900 | 0.2600 | 0.0636 | 0.9603 | 0.1750 | 0.0107 |
| 107 | BF+PL+SO | 0.9112±0.0838 | 0.9724±0.0488 | 4.5474±1.5522 | 0.1174±0.0547 | 0.0900 | 0.3100 | 0.0582 | 0.9191 | 0.2000 | 0.0107 |
| 108 | GMax+RF+SO+TA+VM | 0.9223±0.0545 | 0.9723±0.0596 | 4.4744±1.4844 | 0.1127±0.0579 | 0.1200 | 0.3400 | 0.0527 | 0.8929 | 0.2300 | 0.0108 |
| 109 | BF+PL+RF+SO+TA+VM | 0.9152±0.1135 | 0.9777±0.0246 | 4.3794±1.7147 | 0.1072±0.0478 | 0.0300 | 0.4400 | 0.0536 | 0.8613 | 0.2350 | 0.0108 |
| 110 | BF+GMax+RF+SO+TA+VM | 0.9245±0.0584 | 0.9533±0.1498 | 4.2731±1.4012 | 0.1225±0.0799 | 0.0900 | 0.3000 | 0.0611 | 0.9134 | 0.1950 | 0.0109 |
| 111 | BF+PL+RF+SO | 0.9152±0.0699 | 0.9727±0.0457 | 4.5216±1.5436 | 0.1159±0.0527 | 0.1900 | 0.2400 | 0.0561 | 0.9105 | 0.2150 | 0.0110 |
| 112 | BF+MG+PL+SO+TA | 0.9138±0.1086 | 0.9741±0.0575 | 4.3300±1.6241 | 0.1062±0.0584 | 0.4300 | 0.0300 | 0.0561 | 0.8524 | 0.2300 | 0.0110 |
| 113 | BF+GMax+PL+RF+VM | 0.9121±0.0790 | 0.9664±0.0796 | 4.5633±1.6307 | 0.1205±0.0579 | 0.0600 | 0.3300 | 0.0607 | 0.9333 | 0.1950 | 0.0111 |
| 114 | BF+GMax+PL+SO | 0.9132±0.1004 | 0.9693±0.0625 | 4.6473±1.6099 | 0.1161±0.0586 | 0.3200 | 0.0900 | 0.0588 | 0.9235 | 0.2050 | 0.0111 |
| 115 | BF+MG | 0.9045±0.1200 | 0.9738±0.0613 | 4.6379±1.7870 | 0.1101±0.0598 | 0.0700 | 0.3400 | 0.0608 | 0.8981 | 0.2050 | 0.0112 |
| 116 | BF+GMax+RF+TA | 0.9188±0.0714 | 0.9682±0.0648 | 4.5879±1.7141 | 0.1174±0.0616 | 0.2400 | 0.1900 | 0.0565 | 0.9231 | 0.2150 | 0.0112 |
| 117 | PL+SO | 0.9057±0.0790 | 0.9714±0.0555 | 4.7651±1.5535 | 0.1169±0.0558 | 0.0300 | 0.3600 | 0.0614 | 0.9382 | 0.1950 | 0.0112 |
| 118 | GMax+PL+SO+VM | 0.9121±0.0870 | 0.9679±0.0601 | 4.5963±1.3802 | 0.1217±0.0601 | 0.0500 | 0.3500 | 0.0600 | 0.9414 | 0.2000 | 0.0113 |
| 119 | GMax+PL+RF+SO | 0.9075±0.0729 | 0.9679±0.0817 | 4.6804±1.5037 | 0.1195±0.0579 | 0.0500 | 0.3400 | 0.0623 | 0.9406 | 0.1950 | 0.0114 |
| 120 | GMax+PL+TA+VM | 0.9237±0.0570 | 0.9712±0.0867 | 4.4970±1.3572 | 0.1118±0.0585 | 0.2500 | 0.2400 | 0.0526 | 0.8914 | 0.2450 | 0.0115 |
| 121 | BF+GMax+RF+SO+VM | 0.9120±0.0653 | 0.9642±0.0876 | 4.5707±1.3732 | 0.1193±0.0668 | 0.2300 | 0.1700 | 0.0619 | 0.9292 | 0.2000 | 0.0115 |
| 122 | BF+PL+SO+TA+VM | 0.9037±0.1000 | 0.9720±0.0712 | 4.6591±1.7663 | 0.1108±0.0572 | 0.3300 | 0.0800 | 0.0622 | 0.9030 | 0.2050 | 0.0115 |
| 123 | BF+MG+GMax+SO+TA+VM | 0.9144±0.0761 | 0.9792±0.0316 | 4.4039±1.5695 | 0.1041±0.0571 | 0.3700 | 0.1400 | 0.0532 | 0.8510 | 0.2550 | 0.0115 |
| 124 | MG+GMax+RF+VM | 0.9107±0.0789 | 0.9644±0.1040 | 4.6398±1.6908 | 0.1124±0.0737 | 0.3100 | 0.1100 | 0.0624 | 0.9077 | 0.2100 | 0.0119 |
| 125 | BF+MG+GMax+VM | 0.8951±0.1061 | 0.9730±0.0521 | 4.8059±1.7255 | 0.1099±0.0585 | 0.2400 | 0.1600 | 0.0660 | 0.9135 | 0.2000 | 0.0120 |
| 126 | GMax+TA | 0.9006±0.0819 | 0.9765±0.0222 | 4.8459±1.6783 | 0.1104±0.0433 | 0.1500 | 0.2800 | 0.0615 | 0.9194 | 0.2150 | 0.0121 |
| 127 | PL+RF+TA | 0.9202±0.0699 | 0.9671±0.0721 | 4.4449±1.5511 | 0.1196±0.0598 | 0.0300 | 0.4400 | 0.0563 | 0.9182 | 0.2350 | 0.0122 |
| 128 | BF+GMax+RF+TA+VM | 0.9125±0.0988 | 0.9709±0.0435 | 4.5796±1.6328 | 0.1149±0.0548 | 0.0300 | 0.4300 | 0.0583 | 0.9121 | 0.2300 | 0.0122 |
| 129 | GMax+PL+TA | 0.9138±0.0708 | 0.9771±0.0222 | 4.7428±1.5792 | 0.1118±0.0496 | 0.4900 | 0.0000 | 0.0545 | 0.9152 | 0.2450 | 0.0122 |
| 130 | BF+MG+GMax+PL+TA+VM | 0.9226±0.0650 | 0.9754±0.0419 | 4.2228±1.6730 | 0.1071±0.0538 | 0.0600 | 0.5100 | 0.0510 | 0.8457 | 0.2850 | 0.0123 |
| 131 | BF+MG+GMax+RF+TA+VM | 0.9175±0.0774 | 0.9769±0.0403 | 4.4509±1.4992 | 0.1061±0.0568 | 0.5000 | 0.0400 | 0.0528 | 0.8637 | 0.2700 | 0.0123 |
| 132 | BF+SO | 0.9021±0.0956 | 0.9716±0.0614 | 4.7949±1.6730 | 0.1146±0.0558 | 0.4100 | 0.0100 | 0.0631 | 0.9317 | 0.2100 | 0.0124 |
| 133 | MG+GMax+RF+SO+TA+VM | 0.9138±0.0982 | 0.9752±0.0362 | 4.3504±1.6835 | 0.1072±0.0525 | 0.3700 | 0.1600 | 0.0555 | 0.8584 | 0.2650 | 0.0126 |
| 134 | MG+GMax+PL+RF+VM | 0.9167±0.0703 | 0.9750±0.0417 | 4.5352±1.4417 | 0.1092±0.0583 | 0.2400 | 0.2900 | 0.0541 | 0.8845 | 0.2650 | 0.0127 |
| 135 | SO+TA | 0.9158±0.0643 | 0.9743±0.0422 | 4.6204±1.5791 | 0.1134±0.0522 | 0.1900 | 0.3200 | 0.0549 | 0.9099 | 0.2550 | 0.0127 |
| 136 | MG+GMax+PL+RF+SO+TA+VM | 0.9292±0.0461 | 0.9735±0.0592 | 4.1895±1.3382 | 0.1048±0.0617 | 0.1500 | 0.4800 | 0.0486 | 0.8331 | 0.3150 | 0.0128 |
| 137 | MG+TA | 0.9189±0.0644 | 0.9751±0.0368 | 4.5525±1.4930 | 0.1109±0.0528 | 0.0500 | 0.4900 | 0.0530 | 0.8931 | 0.2700 | 0.0128 |
| 138 | MG+GMax+VM | 0.9122±0.0687 | 0.9761±0.0400 | 4.6217±1.4988 | 0.1077±0.0532 | 0.2600 | 0.2600 | 0.0558 | 0.8867 | 0.2600 | 0.0129 |
| 139 | BF+MG+GMax+PL+RF+SO+TA | 0.9200±0.0782 | 0.9743±0.0471 | 4.2649±1.7018 | 0.1041±0.0599 | 0.4700 | 0.1200 | 0.0528 | 0.8375 | 0.2950 | 0.0131 |
| 140 | TA+VM | 0.9075±0.0725 | 0.9755±0.0250 | 4.7518±1.4289 | 0.1153±0.0493 | 0.1500 | 0.3300 | 0.0585 | 0.9303 | 0.2400 | 0.0131 |
| 141 | BF+MG+GMax+RF+SO+TA | 0.9143±0.0910 | 0.9769±0.0331 | 4.4894±1.7138 | 0.1076±0.0503 | 0.0600 | 0.4900 | 0.0544 | 0.8735 | 0.2750 | 0.0131 |
| 142 | BF+MG+RF+TA | 0.9131±0.0828 | 0.9779±0.0533 | 4.4694±1.7569 | 0.1043±0.0499 | 0.3100 | 0.2500 | 0.0545 | 0.8581 | 0.2800 | 0.0131 |
| 143 | BF+RF+SO | 0.9065±0.0980 | 0.9711±0.0668 | 4.6543±1.7799 | 0.1155±0.0568 | 0.1600 | 0.3100 | 0.0612 | 0.9217 | 0.2350 | 0.0133 |
| 144 | MG+SO | 0.9113±0.0836 | 0.9705±0.0875 | 4.6072±1.5656 | 0.1112±0.0616 | 0.2300 | 0.2700 | 0.0591 | 0.8996 | 0.2500 | 0.0133 |
| 145 | BF+GMax+PL+RF | 0.9022±0.1222 | 0.9710±0.0495 | 4.6566±1.6610 | 0.1135±0.0540 | 0.0700 | 0.3900 | 0.0634 | 0.9138 | 0.2300 | 0.0133 |
| 146 | MG+PL+RF+TA | 0.9193±0.0721 | 0.9648±0.1029 | 4.4062±1.5339 | 0.1166±0.0777 | 0.1100 | 0.4000 | 0.0579 | 0.9022 | 0.2550 | 0.0133 |
| 147 | MG+SO+TA+VM | 0.9166±0.0729 | 0.9763±0.0575 | 4.4269±1.6335 | 0.1067±0.0541 | 0.2300 | 0.3500 | 0.0536 | 0.8638 | 0.2900 | 0.0134 |
| 148 | BF+VM | 0.8981±0.0861 | 0.9727±0.0314 | 4.9269±1.6400 | 0.1170±0.0492 | 0.0700 | 0.3700 | 0.0646 | 0.9542 | 0.2200 | 0.0136 |
| 149 | BF+GMax+RF+SO | 0.9016±0.0859 | 0.9649±0.0829 | 4.7697±1.7526 | 0.1190±0.0641 | 0.0600 | 0.3700 | 0.0667 | 0.9472 | 0.2150 | 0.0136 |
| 150 | MG+VM | 0.9070±0.0781 | 0.9778±0.0459 | 4.6597±1.5829 | 0.1080±0.0550 | 0.2500 | 0.2800 | 0.0576 | 0.8916 | 0.2650 | 0.0136 |
| 151 | MG+GMax+PL+RF+TA+VM | 0.9243±0.0587 | 0.9710±0.0642 | 4.2112±1.4721 | 0.1091±0.0649 | 0.2400 | 0.3700 | 0.0523 | 0.8527 | 0.3050 | 0.0136 |
| 152 | BF+GMax+PL+VM | 0.9092±0.0744 | 0.9716±0.0385 | 4.6980±1.4745 | 0.1185±0.0541 | 0.1700 | 0.3200 | 0.0596 | 0.9382 | 0.2450 | 0.0137 |
| 153 | BF+GMax | 0.8899±0.0921 | 0.9726±0.0265 | 5.0756±1.8003 | 0.1197±0.0472 | 0.0700 | 0.3400 | 0.0687 | 0.9796 | 0.2050 | 0.0138 |
| 154 | BF+GMax+RF+VM | 0.9018±0.0924 | 0.9659±0.0898 | 4.8817±1.6350 | 0.1186±0.0637 | 0.3300 | 0.1100 | 0.0662 | 0.9564 | 0.2200 | 0.0139 |
| 155 | BF+GMax+PL+TA+VM | 0.9242±0.0551 | 0.9763±0.0476 | 4.3350±1.4126 | 0.1086±0.0508 | 0.4600 | 0.1900 | 0.0498 | 0.8627 | 0.3250 | 0.0139 |
| 156 | BF+PL+RF+SO+TA | 0.9150±0.1049 | 0.9708±0.0396 | 4.5093±1.6261 | 0.1149±0.0535 | 0.1200 | 0.4200 | 0.0571 | 0.9053 | 0.2700 | 0.0140 |
| 157 | GMax+PL+RF+TA+VM | 0.9231±0.0543 | 0.9716±0.0456 | 4.4577±1.3696 | 0.1113±0.0554 | 0.4900 | 0.1100 | 0.0527 | 0.8856 | 0.3000 | 0.0140 |
| 158 | BF+PL | 0.9042±0.0794 | 0.9693±0.0737 | 4.7680±1.7157 | 0.1177±0.0572 | 0.0900 | 0.3800 | 0.0633 | 0.9417 | 0.2350 | 0.0140 |
| 159 | BF+GMax+PL+SO+TA+VM | 0.9127±0.0993 | 0.9708±0.0514 | 4.3699±1.6399 | 0.1164±0.0551 | 0.2000 | 0.3400 | 0.0583 | 0.8979 | 0.2700 | 0.0141 |
| 160 | BF+MG+RF+SO | 0.9065±0.0776 | 0.9789±0.0356 | 4.6332±1.6148 | 0.1027±0.0509 | 0.4400 | 0.1300 | 0.0573 | 0.8674 | 0.2850 | 0.0142 |
| 161 | BF+MG+GMax+PL | 0.8995±0.1392 | 0.9675±0.0978 | 4.6550±1.9319 | 0.1120±0.0746 | 0.2800 | 0.1900 | 0.0665 | 0.9075 | 0.2350 | 0.0142 |
| 162 | BF+MG+GMax+PL+RF+TA+VM | 0.9183±0.0677 | 0.9719±0.0780 | 4.3469±1.6194 | 0.1086±0.0608 | 0.2000 | 0.4000 | 0.0549 | 0.8638 | 0.3000 | 0.0142 |
| 163 | GMax+PL+SO | 0.9138±0.0661 | 0.9739±0.0268 | 4.6431±1.4773 | 0.1162±0.0491 | 0.2100 | 0.3400 | 0.0561 | 0.9235 | 0.2750 | 0.0143 |
| 164 | PL+RF+SO+TA | 0.9141±0.0701 | 0.9748±0.0283 | 4.4986±1.6286 | 0.1123±0.0496 | 0.2300 | 0.3500 | 0.0556 | 0.8936 | 0.2900 | 0.0144 |
| 165 | GMax+PL | 0.9015±0.0814 | 0.9753±0.0259 | 4.9124±1.6373 | 0.1142±0.0503 | 0.2000 | 0.3000 | 0.0616 | 0.9414 | 0.2500 | 0.0145 |
| 166 | PL+RF+SO+VM | 0.9145±0.0635 | 0.9703±0.0447 | 4.5870±1.3195 | 0.1205±0.0513 | 0.0500 | 0.4900 | 0.0576 | 0.9356 | 0.2700 | 0.0146 |
| 167 | GMax+SO+TA+VM | 0.9161±0.0644 | 0.9744±0.0466 | 4.5157±1.4745 | 0.1115±0.0532 | 0.1300 | 0.4700 | 0.0547 | 0.8920 | 0.3000 | 0.0147 |
| 168 | RF+SO+VM | 0.9091±0.0700 | 0.9751±0.0272 | 4.7473±1.4916 | 0.1135±0.0464 | 0.1200 | 0.4300 | 0.0579 | 0.9226 | 0.2750 | 0.0147 |
| 169 | PL+RF+VM | 0.9129±0.0668 | 0.9733±0.0354 | 4.6622±1.4130 | 0.1167±0.0524 | 0.0500 | 0.5100 | 0.0569 | 0.9274 | 0.2800 | 0.0148 |
| 170 | MG+PL+VM | 0.9096±0.0793 | 0.9727±0.0671 | 4.6637±1.4549 | 0.1137±0.0609 | 0.1000 | 0.4500 | 0.0588 | 0.9153 | 0.2750 | 0.0148 |
| 171 | BF+MG+RF+SO+VM | 0.9139±0.0770 | 0.9777±0.0341 | 4.3484±1.6822 | 0.1108±0.0554 | 0.0900 | 0.5400 | 0.0542 | 0.8729 | 0.3150 | 0.0149 |
| 172 | MG+PL+SO+VM | 0.9148±0.0681 | 0.9769±0.0269 | 4.4736±1.5134 | 0.1093±0.0500 | 0.1100 | 0.5200 | 0.0542 | 0.8789 | 0.3150 | 0.0150 |
| 173 | PL+RF+TA+VM | 0.9234±0.0619 | 0.9601±0.1179 | 4.4455±1.3774 | 0.1168±0.0712 | 0.4700 | 0.1000 | 0.0583 | 0.9068 | 0.2850 | 0.0151 |
| 174 | MG+PL+RF+SO+VM | 0.9126±0.0781 | 0.9767±0.0341 | 4.4760±1.4633 | 0.1094±0.0506 | 0.2300 | 0.3900 | 0.0554 | 0.8796 | 0.3100 | 0.0151 |
| 175 | GMax+TA+VM | 0.9090±0.0824 | 0.9714±0.0381 | 4.6330±1.5348 | 0.1182±0.0522 | 0.1700 | 0.3800 | 0.0598 | 0.9307 | 0.2750 | 0.0153 |
| 176 | BF | 0.8809±0.1277 | 0.9736±0.0264 | 5.1351±2.0436 | 0.1181±0.0471 | 0.1800 | 0.2500 | 0.0728 | 0.9789 | 0.2150 | 0.0153 |
| 177 | BF+MG+RF | 0.9039±0.1272 | 0.9781±0.0303 | 4.6485±1.7015 | 0.1069±0.0490 | 0.3900 | 0.2000 | 0.0590 | 0.8861 | 0.2950 | 0.0154 |
| 178 | BF+RF+VM | 0.8948±0.0900 | 0.9724±0.0394 | 4.9445±1.7071 | 0.1157±0.0514 | 0.2600 | 0.2300 | 0.0664 | 0.9506 | 0.2450 | 0.0155 |
| 179 | BF+GMax+PL+RF+SO+TA+VM | 0.9196±0.0731 | 0.9681±0.0706 | 4.4045±1.6198 | 0.1169±0.0613 | 0.1400 | 0.4700 | 0.0561 | 0.9033 | 0.3050 | 0.0155 |
| 180 | BF+GMax+PL+RF+SO | 0.9082±0.1023 | 0.9723±0.0446 | 4.6203±1.5634 | 0.1159±0.0554 | 0.1800 | 0.3900 | 0.0597 | 0.9201 | 0.2850 | 0.0157 |
| 181 | MG+RF+SO+VM | 0.9136±0.0758 | 0.9777±0.0338 | 4.5355±1.4879 | 0.1051±0.0495 | 0.2300 | 0.4400 | 0.0543 | 0.8678 | 0.3350 | 0.0158 |
| 182 | BF+SO+VM | 0.9086±0.0838 | 0.9695±0.0598 | 4.6771±1.6510 | 0.1181±0.0560 | 0.0800 | 0.4900 | 0.0610 | 0.9345 | 0.2850 | 0.0162 |
| 183 | BF+MG+TA+VM | 0.9235±0.0634 | 0.9808±0.0242 | 4.3637±1.4577 | 0.1022±0.0463 | 0.4000 | 0.4100 | 0.0479 | 0.8393 | 0.4050 | 0.0163 |
| 184 | MG+RF+TA+VM | 0.9185±0.0646 | 0.9722±0.0716 | 4.4456±1.5105 | 0.1128±0.0677 | 0.1100 | 0.5600 | 0.0547 | 0.8905 | 0.3350 | 0.0163 |
| 185 | BF+GMax+PL+TA | 0.8991±0.0889 | 0.9676±0.0756 | 4.7726±1.6655 | 0.1142±0.0608 | 0.3000 | 0.2300 | 0.0667 | 0.9279 | 0.2650 | 0.0164 |
| 186 | BF+MG+GMax+TA | 0.9073±0.0839 | 0.9778±0.0265 | 4.4505±1.6480 | 0.1081±0.0481 | 0.2100 | 0.4500 | 0.0574 | 0.8718 | 0.3300 | 0.0165 |
| 187 | RF | 0.8871±0.0837 | 0.9746±0.0256 | 5.0989±1.7958 | 0.1146±0.0428 | 0.3100 | 0.1900 | 0.0692 | 0.9611 | 0.2500 | 0.0166 |
| 188 | MG+GMax+SO | 0.9036±0.0854 | 0.9731±0.0561 | 4.7810±1.7084 | 0.1121±0.0607 | 0.2500 | 0.3400 | 0.0617 | 0.9201 | 0.2950 | 0.0167 |
| 189 | MG | 0.8993±0.0871 | 0.9781±0.0316 | 4.7901±1.8026 | 0.1105±0.0486 | 0.0500 | 0.5500 | 0.0613 | 0.9145 | 0.3000 | 0.0168 |
| 190 | PL+RF+SO | 0.9050±0.0830 | 0.9748±0.0311 | 4.7897±1.6120 | 0.1164±0.0473 | 0.1400 | 0.4600 | 0.0601 | 0.9385 | 0.3000 | 0.0169 |
| 191 | GMax+RF+VM | 0.8991±0.0861 | 0.9681±0.0449 | 4.9157±1.6088 | 0.1201±0.0508 | 0.1200 | 0.4100 | 0.0664 | 0.9658 | 0.2650 | 0.0170 |
| 192 | MG+GMax+PL+SO | 0.9092±0.0864 | 0.9622±0.0951 | 4.6712±1.5654 | 0.1168±0.0727 | 0.0800 | 0.4900 | 0.0643 | 0.9287 | 0.2850 | 0.0170 |
| 193 | GMax+RF+SO+TA | 0.9156±0.0849 | 0.9692±0.0650 | 4.6700±1.8370 | 0.1165±0.0579 | 0.2600 | 0.3800 | 0.0576 | 0.9273 | 0.3200 | 0.0171 |
| 194 | GMax+RF+SO+VM | 0.9123±0.0634 | 0.9697±0.0503 | 4.6599±1.5152 | 0.1165±0.0530 | 0.0300 | 0.6000 | 0.0590 | 0.9264 | 0.3150 | 0.0172 |
| 195 | BF+GMax+PL+SO+TA | 0.9163±0.0721 | 0.9715±0.0360 | 4.4266±1.6149 | 0.1168±0.0514 | 0.1700 | 0.5100 | 0.0561 | 0.9050 | 0.3400 | 0.0173 |
| 196 | BF+SO+TA+VM | 0.9135±0.0689 | 0.9703±0.0686 | 4.6599±1.5420 | 0.1125±0.0581 | 0.5900 | 0.0700 | 0.0581 | 0.9100 | 0.3300 | 0.0174 |
| 197 | BF+MG+GMax+PL+RF+SO+VM | 0.9005±0.0940 | 0.9803±0.0262 | 4.5170±1.8822 | 0.1021±0.0458 | 0.2900 | 0.4000 | 0.0596 | 0.8538 | 0.3450 | 0.0176 |
| 198 | BF+PL+RF+TA+VM | 0.9170±0.0871 | 0.9688±0.0565 | 4.5476±1.5923 | 0.1173±0.0584 | 0.2600 | 0.4100 | 0.0571 | 0.9188 | 0.3350 | 0.0176 |
| 199 | TA | 0.8998±0.0859 | 0.9750±0.0247 | 4.8687±1.8073 | 0.1149±0.0442 | 0.2200 | 0.3900 | 0.0626 | 0.9400 | 0.3050 | 0.0179 |
| 200 | PL+SO+TA | 0.9078±0.1049 | 0.9723±0.0640 | 4.6312±1.7722 | 0.1156±0.0582 | 0.5800 | 0.0800 | 0.0599 | 0.9199 | 0.3300 | 0.0182 |
| 201 | BF+GMax+SO+TA | 0.9201±0.0614 | 0.9603±0.1150 | 4.3864±1.5150 | 0.1195±0.0707 | 0.0500 | 0.6200 | 0.0598 | 0.9121 | 0.3350 | 0.0183 |
| 202 | MG+PL+RF | 0.8999±0.0978 | 0.9761±0.0355 | 4.7546±1.5539 | 0.1113±0.0567 | 0.1600 | 0.4900 | 0.0620 | 0.9143 | 0.3250 | 0.0184 |
| 203 | BF+PL+RF+SO+VM | 0.9125±0.0761 | 0.9710±0.0533 | 4.4740±1.5432 | 0.1159±0.0620 | 0.2600 | 0.4400 | 0.0582 | 0.9059 | 0.3500 | 0.0185 |
| 204 | MG+RF+SO | 0.9119±0.0668 | 0.9736±0.0307 | 4.5912±1.6210 | 0.1139±0.0487 | 0.0700 | 0.6400 | 0.0573 | 0.9091 | 0.3550 | 0.0185 |
| 205 | MG+GMax+PL+SO+TA | 0.9214±0.0694 | 0.9743±0.0349 | 4.3686±1.5525 | 0.1125±0.0557 | 0.3800 | 0.4300 | 0.0521 | 0.8818 | 0.4050 | 0.0186 |
| 206 | PL+RF | 0.9019±0.0753 | 0.9736±0.0334 | 4.8592±1.7066 | 0.1156±0.0467 | 0.0800 | 0.5600 | 0.0623 | 0.9420 | 0.3200 | 0.0188 |
| 207 | BF+MG+PL+RF+TA | 0.9129±0.0936 | 0.9729±0.0482 | 4.3756±1.5747 | 0.1063±0.0583 | 0.1200 | 0.6500 | 0.0571 | 0.8572 | 0.3850 | 0.0188 |
| 208 | VM | 0.8953±0.0842 | 0.9726±0.0263 | 4.9771±1.7032 | 0.1194±0.0455 | 0.0600 | 0.5300 | 0.0661 | 0.9689 | 0.2950 | 0.0189 |
| 209 | MG+GMax+SO+TA+VM | 0.9194±0.0523 | 0.9764±0.0328 | 4.4658±1.5844 | 0.1068±0.0548 | 0.2700 | 0.5700 | 0.0521 | 0.8680 | 0.4200 | 0.0190 |
| 210 | BF+MG+PL+RF+SO+TA+VM | 0.9192±0.0718 | 0.9731±0.0869 | 4.3243±1.4617 | 0.1041±0.0600 | 0.5900 | 0.2500 | 0.0538 | 0.8433 | 0.4200 | 0.0191 |
| 211 | BF+MG+SO | 0.8959±0.0895 | 0.9769±0.0457 | 4.8227±1.6802 | 0.1075±0.0516 | 0.4700 | 0.2100 | 0.0636 | 0.9054 | 0.3400 | 0.0196 |
| 212 | BF+GMax+RF+SO+TA | 0.9086±0.1020 | 0.9707±0.0702 | 4.5512±1.9350 | 0.1134±0.0588 | 0.3000 | 0.4200 | 0.0604 | 0.9032 | 0.3600 | 0.0196 |
| 213 | MG+GMax+TA+VM | 0.9084±0.0798 | 0.9761±0.0438 | 4.5561±1.5314 | 0.1092±0.0545 | 0.4900 | 0.2800 | 0.0577 | 0.8865 | 0.3850 | 0.0197 |
| 214 | BF+RF+TA+VM | 0.9191±0.0581 | 0.9745±0.0348 | 4.4867±1.4495 | 0.1160±0.0548 | 0.2900 | 0.5300 | 0.0532 | 0.9076 | 0.4100 | 0.0198 |
| 215 | BF+GMax+SO+TA+VM | 0.9183±0.0567 | 0.9686±0.0598 | 4.4643±1.5228 | 0.1172±0.0573 | 0.3500 | 0.4200 | 0.0566 | 0.9103 | 0.3850 | 0.0198 |
| 216 | BF+GMax+TA+VM | 0.9146±0.0796 | 0.9662±0.0726 | 4.6088±1.5445 | 0.1175±0.0596 | 0.2700 | 0.4500 | 0.0596 | 0.9255 | 0.3600 | 0.0199 |
| 217 | MG+PL+TA+VM | 0.9225±0.0593 | 0.9802±0.0243 | 4.3471±1.4348 | 0.1018±0.0437 | 0.4700 | 0.5100 | 0.0486 | 0.8361 | 0.4900 | 0.0199 |
| 218 | PL+TA | 0.9140±0.0666 | 0.9768±0.0302 | 4.7066±1.5101 | 0.1125±0.0479 | 0.3000 | 0.5000 | 0.0546 | 0.9145 | 0.4000 | 0.0200 |
| 219 | BF+GMax+PL+RF+SO+TA | 0.9158±0.0683 | 0.9722±0.0397 | 4.4553±1.5759 | 0.1131±0.0522 | 0.3800 | 0.4200 | 0.0560 | 0.8927 | 0.4000 | 0.0200 |
| 220 | BF+MG+GMax+PL+RF+TA | 0.9142±0.0743 | 0.9709±0.0462 | 4.4338±1.6536 | 0.1144±0.0598 | 0.3500 | 0.4300 | 0.0574 | 0.8959 | 0.3900 | 0.0201 |
| 221 | BF+MG+GMax+SO+TA | 0.9126±0.0866 | 0.9734±0.0602 | 4.5817±1.7137 | 0.1083±0.0586 | 0.6500 | 0.1500 | 0.0570 | 0.8853 | 0.4000 | 0.0202 |
| 222 | BF+SO+TA | 0.9201±0.0682 | 0.9666±0.0855 | 4.4315±1.6033 | 0.1190±0.0713 | 0.2600 | 0.5200 | 0.0567 | 0.9145 | 0.3900 | 0.0202 |
| 223 | BF+GMax+PL+SO+VM | 0.9095±0.0791 | 0.9694±0.0663 | 4.5912±1.5458 | 0.1157±0.0589 | 0.2300 | 0.5000 | 0.0606 | 0.9164 | 0.3650 | 0.0203 |
| 224 | RF+SO | 0.9059±0.0705 | 0.9762±0.0229 | 4.8538±1.5190 | 0.1140±0.0439 | 0.2800 | 0.4800 | 0.0590 | 0.9349 | 0.3800 | 0.0209 |
| 225 | BF+PL+VM | 0.9109±0.0741 | 0.9740±0.0329 | 4.6526±1.5321 | 0.1163±0.0495 | 0.1500 | 0.6500 | 0.0576 | 0.9248 | 0.4000 | 0.0213 |
| 226 | BF+GMax+PL | 0.9083±0.0720 | 0.9703±0.0769 | 4.7530±1.5238 | 0.1186±0.0575 | 0.4500 | 0.3000 | 0.0607 | 0.9439 | 0.3750 | 0.0215 |
| 227 | BF+MG+PL+TA | 0.9195±0.0833 | 0.9758±0.0311 | 4.3042±1.6936 | 0.1110±0.0533 | 0.4500 | 0.5100 | 0.0524 | 0.8695 | 0.4800 | 0.0218 |
| 228 | GMax+PL+RF+SO+TA | 0.9195±0.0608 | 0.9710±0.0418 | 4.4721±1.5847 | 0.1169±0.0571 | 0.4700 | 0.4100 | 0.0547 | 0.9098 | 0.4400 | 0.0219 |
| 229 | MG+GMax+PL+RF+SO | 0.9042±0.0940 | 0.9749±0.0339 | 4.8711±1.7512 | 0.1101±0.0561 | 0.7600 | 0.0400 | 0.0604 | 0.9207 | 0.4000 | 0.0223 |
| 230 | BF+MG+GMax+PL+RF | 0.9128±0.0715 | 0.9699±0.0588 | 4.5079±1.5161 | 0.1139±0.0666 | 0.3300 | 0.5200 | 0.0586 | 0.9010 | 0.4250 | 0.0225 |
| 231 | BF+MG+GMax+RF | 0.9012±0.0962 | 0.9657±0.1154 | 4.6182±1.6864 | 0.1111±0.0707 | 0.5500 | 0.2000 | 0.0666 | 0.9003 | 0.3750 | 0.0225 |
| 232 | PL+SO+VM | 0.9165±0.0616 | 0.9777±0.0218 | 4.6493±1.3426 | 0.1118±0.0425 | 0.4800 | 0.4600 | 0.0529 | 0.9061 | 0.4700 | 0.0225 |
| 233 | MG+PL+RF+SO | 0.9075±0.0730 | 0.9706±0.0777 | 4.6334±1.5433 | 0.1075±0.0661 | 0.5800 | 0.2600 | 0.0609 | 0.8871 | 0.4200 | 0.0227 |
| 234 | BF+RF | 0.8938±0.0794 | 0.9735±0.0296 | 5.0281±1.6662 | 0.1170±0.0476 | 0.2800 | 0.4500 | 0.0663 | 0.9640 | 0.3650 | 0.0233 |
| 235 | MG+GMax+PL | 0.8927±0.1190 | 0.9750±0.0322 | 4.9577±1.8940 | 0.1141±0.0556 | 0.4500 | 0.3200 | 0.0661 | 0.9454 | 0.3850 | 0.0241 |
| 236 | BF+RF+SO+VM | 0.9056±0.0850 | 0.9688±0.0720 | 4.7433±1.7910 | 0.1169±0.0602 | 0.4700 | 0.3500 | 0.0628 | 0.9361 | 0.4100 | 0.0241 |
| 237 | MG+SO+VM | 0.9091±0.0725 | 0.9799±0.0222 | 4.6322±1.5018 | 0.1097±0.0540 | 0.4000 | 0.5700 | 0.0555 | 0.8959 | 0.4850 | 0.0241 |
| 238 | BF+MG+PL+RF+SO+VM | 0.9172±0.0814 | 0.9715±0.0598 | 4.4573±1.5345 | 0.1118±0.0597 | 0.5000 | 0.4800 | 0.0556 | 0.8876 | 0.4900 | 0.0242 |
| 239 | BF+MG+GMax+SO+VM | 0.9089±0.0682 | 0.9767±0.0482 | 4.6934±1.4973 | 0.1096±0.0628 | 0.6300 | 0.3500 | 0.0572 | 0.9014 | 0.4900 | 0.0253 |
| 240 | BF+MG+GMax+PL+SO | 0.9026±0.1091 | 0.9731±0.0485 | 4.7924±1.8554 | 0.1119±0.0634 | 0.6600 | 0.2300 | 0.0622 | 0.9204 | 0.4450 | 0.0255 |
| 241 | BF+PL+SO+TA | 0.9102±0.1152 | 0.9704±0.0424 | 4.5975±1.7209 | 0.1182±0.0560 | 0.5500 | 0.3900 | 0.0597 | 0.9273 | 0.4700 | 0.0260 |
| 242 | BF+PL+TA | 0.9196±0.0660 | 0.9737±0.0523 | 4.5939±1.4682 | 0.1143±0.0570 | 0.5600 | 0.5200 | 0.0534 | 0.9110 | 0.5400 | 0.0262 |
| 243 | GMax+PL+SO+TA | 0.9133±0.0778 | 0.9735±0.0299 | 4.6736±1.5800 | 0.1158±0.0522 | 0.5100 | 0.5500 | 0.0566 | 0.9248 | 0.5300 | 0.0277 |
| 244 | MG+GMax+RF+TA+VM | 0.9082±0.1124 | 0.9793±0.0237 | 4.5153±1.6160 | 0.1044±0.0473 | 0.5600 | 0.5900 | 0.0562 | 0.8630 | 0.5750 | 0.0279 |
| 245 | BF+PL+RF | 0.8929±0.1319 | 0.9757±0.0297 | 4.8344±1.8748 | 0.1146±0.0471 | 0.3100 | 0.6200 | 0.0657 | 0.9355 | 0.4650 | 0.0286 |
| 246 | MG+PL+SO+TA | 0.9121±0.0902 | 0.9732±0.0621 | 4.4892±1.6541 | 0.1118±0.0583 | 0.5300 | 0.6000 | 0.0573 | 0.8907 | 0.5650 | 0.0289 |
| 247 | RF+VM | 0.9009±0.0784 | 0.9712±0.0451 | 4.8933±1.6459 | 0.1181±0.0551 | 0.3900 | 0.5800 | 0.0640 | 0.9555 | 0.4850 | 0.0296 |
| 248 | BF+GMax+PL+RF+SO+VM | 0.9075±0.0784 | 0.9686±0.0627 | 4.7066±1.5422 | 0.1138±0.0582 | 0.5700 | 0.5200 | 0.0619 | 0.9198 | 0.5450 | 0.0311 |
| 249 | RF+TA+VM | 0.8905±0.0839 | 0.9717±0.0485 | 4.9624±1.5798 | 0.1169±0.0569 | 0.3600 | 0.5900 | 0.0689 | 0.9573 | 0.4750 | 0.0313 |
| 250 | BF+MG+PL+RF+TA+VM | 0.8889±0.1065 | 0.9724±0.0640 | 4.8497±1.7564 | 0.1097±0.0637 | 0.7000 | 0.3200 | 0.0694 | 0.9170 | 0.5100 | 0.0324 |
| 251 | BF+MG+PL+SO | 0.9120±0.0907 | 0.9705±0.0652 | 4.5136±1.5979 | 0.1146±0.0597 | 0.5100 | 0.7400 | 0.0588 | 0.9044 | 0.6250 | 0.0332 |
| 252 | BF+MG+PL | 0.9065±0.0814 | 0.9698±0.0405 | 4.5834±1.5653 | 0.1201±0.0566 | 0.3200 | 0.8400 | 0.0619 | 0.9336 | 0.5800 | 0.0335 |
| 253 | BF+MG+PL+SO+TA+VM | 0.9076±0.0709 | 0.9744±0.0574 | 4.8778±1.6732 | 0.1088±0.0568 | 0.8800 | 0.4300 | 0.0590 | 0.9160 | 0.6550 | 0.0354 |
| 254 | MG+GMax+PL+SO+TA+VM | 0.9119±0.0738 | 0.9654±0.1053 | 4.7634±1.6304 | 0.1110±0.0714 | 0.7600 | 0.6000 | 0.0614 | 0.9139 | 0.6800 | 0.0381 |
| 255 | BF+MG+GMax+PL+RF+VM | 0.8988±0.0835 | 0.9655±0.0793 | 4.7963±1.5167 | 0.1195±0.0640 | 0.7200 | 0.7700 | 0.0678 | 0.9518 | 0.7450 | 0.0481 |
