# Supplementary material for: The use of nonnormalized surface EMG and feature inputs for LSTM-based powered ankle prosthesis control algorithm development
Source: Front Neurosci. 2023 Jul 3;17:1158280. doi: 10.3389/fnins.2023.1158280 (PMC10351874; doi:10.3389/fnins.2023.1158280)
Supplement: Supplementary file 3 [file Data_Sheet_3.DOCX]

**Table SD3-1.** The comparison of the sEMG feature variations calculated over MG+TA. Green shaded cells show the feature variations that provide strongly correlated position and moment predictions (i.e., r > 0.90). MG: medial gastrocnemius, TA: tibialis anterior, IEMG: integrated EMG, MAV: mean absolute value, WAMP: Willison amplitude, RMS: root mean square; WL: waveform length.

| **Rank** | **sEMG Feature Variations** | **Position Correlation  [r]** | **Moment Correlation  [r]** | **Position RMSE  [deg]** | **Moment RMSE  [Nm/kg]** | **Position SPM [%GC]** | **Moment SPM [%GC]** | **Miscorrelation Score** | **Error  Score** | **SPM Score** | **Overall Error Score** |
| --- | --- | --- | --- | --- | --- | --- | --- | --- | --- | --- | --- |
| 1 | WL | 0.8894±0.0812 | 0.9789±0.0306 | 5.0765±1.6594 | 0.1059±0.0487 | 0.1100 | 0.0300 | 0.0658 | 0.6483 | 0.0700 | 0.0030 |
| 2 | RMS+WL | 0.8875±0.0801 | 0.9767±0.0379 | 5.1379±1.6417 | 0.1100±0.0514 | 0.1400 | 0.1800 | 0.0679 | 0.6660 | 0.1600 | 0.0072 |
| 3 | MAV+RMS+WL | 0.8990±0.0700 | 0.9757±0.0364 | 4.9453±1.5996 | 0.1122±0.0533 | 0.0500 | 0.3500 | 0.0626 | 0.6632 | 0.2000 | 0.0083 |
| 4 | IEMG+RMS+WL | 0.8949±0.0839 | 0.9765±0.0442 | 4.9346±1.5971 | 0.1092±0.0522 | 0.0700 | 0.3400 | 0.0643 | 0.6521 | 0.2050 | 0.0086 |
| 5 | IEMG+WL | 0.9189±0.0644 | 0.9751±0.0368 | 4.5525±1.4930 | 0.1109±0.0528 | 0.0500 | 0.4900 | 0.0530 | 0.6371 | 0.2700 | 0.0091 |
| 6 | IEMG+MAV+RMS+WAMP | 0.9138±0.0718 | 0.9652±0.0904 | 4.5818±1.5335 | 0.1260±0.0634 | 0.1000 | 0.3400 | 0.0605 | 0.6915 | 0.2200 | 0.0092 |
| 7 | WAMP | 0.9154±0.0693 | 0.9744±0.0416 | 4.6681±1.7723 | 0.1159±0.0518 | 0.1500 | 0.4100 | 0.0551 | 0.6609 | 0.2800 | 0.0102 |
| 8 | MAV+RMS+WAMP+WL | 0.9144±0.0707 | 0.9617±0.0650 | 4.5990±1.6603 | 0.1351±0.0607 | 0.1000 | 0.4000 | 0.0619 | 0.7242 | 0.2500 | 0.0112 |
| 9 | RMS+WAMP+WL | 0.9031±0.0894 | 0.9709±0.0474 | 4.7727±1.8347 | 0.1184±0.0577 | 0.3200 | 0.2200 | 0.0630 | 0.6754 | 0.2700 | 0.0115 |
| 10 | IEMG+RMS+WAMP+WL | 0.9126±0.0614 | 0.9723±0.0419 | 4.5823±1.5377 | 0.1186±0.0551 | 0.1100 | 0.4900 | 0.0576 | 0.6656 | 0.3000 | 0.0115 |
| 11 | MAV+RMS+WAMP | 0.9125±0.0727 | 0.9746±0.0264 | 4.6416±1.7388 | 0.1183±0.0464 | 0.1400 | 0.5000 | 0.0564 | 0.6678 | 0.3200 | 0.0121 |
| 12 | IEMG+MAV+WL | 0.8962±0.0820 | 0.9740±0.0457 | 4.9484±1.4857 | 0.1117±0.0539 | 0.3600 | 0.2100 | 0.0649 | 0.6616 | 0.2850 | 0.0122 |
| 13 | IEMG | 0.8742±0.1021 | 0.9776±0.0255 | 5.3140±1.7934 | 0.1097±0.0450 | 0.3700 | 0.1400 | 0.0741 | 0.6746 | 0.2550 | 0.0127 |
| 14 | MAV+WAMP | 0.9168±0.0669 | 0.9711±0.0411 | 4.6124±1.6694 | 0.1205±0.0540 | 0.0900 | 0.5900 | 0.0561 | 0.6739 | 0.3400 | 0.0128 |
| 15 | WAMP+WL | 0.9118±0.0702 | 0.9681±0.0558 | 4.6735±1.5879 | 0.1181±0.0599 | 0.2500 | 0.3900 | 0.0600 | 0.6689 | 0.3200 | 0.0129 |
| 16 | RMS+WAMP | 0.9105±0.0759 | 0.9729±0.0368 | 4.7046±1.7422 | 0.1184±0.0546 | 0.1100 | 0.5600 | 0.0583 | 0.6716 | 0.3350 | 0.0131 |
| 17 | IEMG+MAV+RMS | 0.8856±0.0798 | 0.9772±0.0263 | 5.2000±1.7807 | 0.1112±0.0447 | 0.2500 | 0.3300 | 0.0686 | 0.6736 | 0.2900 | 0.0134 |
| 18 | MAV+WL | 0.8981±0.0694 | 0.9761±0.0331 | 4.9808±1.5676 | 0.1111±0.0523 | 0.1600 | 0.5000 | 0.0629 | 0.6613 | 0.3300 | 0.0137 |
| 19 | MAV+WAMP+WL | 0.9161±0.0650 | 0.9568±0.0600 | 4.6171±1.6292 | 0.1431±0.0612 | 0.1900 | 0.4000 | 0.0635 | 0.7531 | 0.2950 | 0.0141 |
| 20 | IEMG+MAV | 0.8796±0.0874 | 0.9758±0.0312 | 5.2595±1.8340 | 0.1101±0.0514 | 0.0600 | 0.5500 | 0.0723 | 0.6731 | 0.3050 | 0.0148 |
| 21 | IEMG+MAV+RMS+WL | 0.9020±0.0706 | 0.9658±0.0663 | 4.8555±1.5233 | 0.1218±0.0623 | 0.2200 | 0.5400 | 0.0661 | 0.6918 | 0.3800 | 0.0174 |
| 22 | IEMG+MAV+RMS+WAMP+WL | 0.9134±0.0681 | 0.9642±0.0803 | 4.6012±1.5661 | 0.1246±0.0634 | 0.2200 | 0.6100 | 0.0612 | 0.6876 | 0.4150 | 0.0175 |
| 23 | IEMG+MAV+WAMP+WL | 0.9104±0.0771 | 0.9638±0.1084 | 4.6046±1.5920 | 0.1249±0.0684 | 0.3500 | 0.5200 | 0.0629 | 0.6889 | 0.4350 | 0.0188 |
| 24 | IEMG+WAMP+WL | 0.9168±0.0776 | 0.9657±0.0929 | 4.6832±1.6131 | 0.1231±0.0595 | 0.5900 | 0.3700 | 0.0588 | 0.6869 | 0.4800 | 0.0194 |
| 25 | IEMG+WAMP | 0.9034±0.0816 | 0.9726±0.0719 | 4.8223±1.7365 | 0.1135±0.0560 | 0.5200 | 0.4300 | 0.0620 | 0.6610 | 0.4750 | 0.0195 |
| 26 | IEMG+RMS | 0.8851±0.0832 | 0.9726±0.0351 | 5.1959±1.7517 | 0.1167±0.0494 | 0.1100 | 0.7400 | 0.0712 | 0.6926 | 0.4250 | 0.0209 |
| 27 | IEMG+MAV+WAMP | 0.9114±0.0712 | 0.9748±0.0273 | 4.6971±1.7189 | 0.1229±0.0525 | 0.2400 | 0.8500 | 0.0569 | 0.6869 | 0.5450 | 0.0213 |
| 28 | IEMG+RMS+WAMP | 0.8928±0.1018 | 0.9716±0.0626 | 4.8437±1.8153 | 0.1171±0.0566 | 0.5100 | 0.4500 | 0.0678 | 0.6747 | 0.4800 | 0.0220 |
| 29 | MAV+RMS | 0.8314±0.1218 | 0.9759±0.0252 | 5.8084±2.0357 | 0.1138±0.0442 | 0.7300 | 0.3700 | 0.0964 | 0.7161 | 0.5500 | 0.0379 |
| 30 | RMS | 0.2477±0.2939 | 0.9760±0.0249 | 9.1197±2.1572 | 0.1136±0.0441 | 0.8500 | 0.1800 | 0.3882 | 0.8969 | 0.5150 | 0.1793 |
| 31 | MAV | 0.2502±0.2689 | 0.9746±0.0267 | 9.0872±2.1578 | 0.1163±0.0450 | 0.8500 | 0.2600 | 0.3876 | 0.9046 | 0.5550 | 0.1946 |

**Table SD3-2.** The comparison of the sEMG feature variations calculated over BF+MG+TA. Green shaded cells show the feature variations that provide strongly correlated position and moment predictions (i.e., r > 0.90). BF: biceps femoris, MG: medial gastrocnemius, TA: tibialis anterior, IEMG: integrated EMG, MAV: mean absolute value, WAMP: Willison amplitude, RMS: root mean square; WL: waveform length.

| **Rank** | **sEMG Feature Variations** | **Position Correlation  [r]** | **Moment Correlation  [r]** | **Position RMSE  [deg]** | **Moment RMSE  [Nm/kg]** | **Position SPM [%GC]** | **Moment SPM [%GC]** | **Miscorrelation Score** | **Error  Score** | **SPM Score** | **Overall Error Score** |
| --- | --- | --- | --- | --- | --- | --- | --- | --- | --- | --- | --- |
| 1 | IEMG+WL | 0.9199±0.0682 | 0.9787±0.0306 | 4.4577±1.5804 | 0.1028±0.0494 | 0.1400 | 0.2700 | 0.0507 | 0.7654 | 0.2050 | 0.0080 |
| 2 | WL | 0.8889±0.1115 | 0.9779±0.0293 | 5.0233±1.7878 | 0.1075±0.0477 | 0.1100 | 0.2100 | 0.0666 | 0.8326 | 0.1600 | 0.0089 |
| 3 | RMS | 0.8958±0.0942 | 0.9773±0.0230 | 5.0087±1.9647 | 0.1119±0.0435 | 0.0500 | 0.2900 | 0.0635 | 0.8471 | 0.1700 | 0.0091 |
| 4 | MAV | 0.8938±0.0906 | 0.9769±0.0238 | 5.0418±1.9141 | 0.1140±0.0439 | 0.0200 | 0.3200 | 0.0646 | 0.8575 | 0.1700 | 0.0094 |
| 5 | RMS+WL | 0.8930±0.1083 | 0.9806±0.0303 | 4.9612±1.7633 | 0.1021±0.0472 | 0.1400 | 0.2300 | 0.0632 | 0.8078 | 0.1850 | 0.0094 |
| 6 | IEMG+MAV | 0.8876±0.0875 | 0.9713±0.0722 | 5.1315±1.7523 | 0.1144±0.0549 | 0.1300 | 0.2000 | 0.0705 | 0.8670 | 0.1650 | 0.0101 |
| 7 | WAMP+WL | 0.9159±0.0572 | 0.9732±0.0374 | 4.5868±1.6020 | 0.1160±0.0552 | 0.2100 | 0.2700 | 0.0554 | 0.8241 | 0.2400 | 0.0110 |
| 8 | IEMG+MAV+WAMP | 0.9166±0.0644 | 0.9689±0.0424 | 4.5150±1.4903 | 0.1233±0.0570 | 0.1200 | 0.3800 | 0.0572 | 0.8438 | 0.2500 | 0.0121 |
| 9 | IEMG | 0.9086±0.0751 | 0.9783±0.0383 | 4.7683±1.6969 | 0.1067±0.0516 | 0.2700 | 0.2900 | 0.0566 | 0.8070 | 0.2800 | 0.0128 |
| 10 | IEMG+WAMP | 0.9113±0.0772 | 0.9712±0.0401 | 4.6174±1.6718 | 0.1193±0.0588 | 0.2600 | 0.2600 | 0.0588 | 0.8387 | 0.2600 | 0.0128 |
| 11 | IEMG+MAV+WL | 0.9075±0.0743 | 0.9747±0.0462 | 4.7171±1.4666 | 0.1087±0.0554 | 0.1900 | 0.3800 | 0.0589 | 0.8096 | 0.2850 | 0.0136 |
| 12 | MAV+RMS+WAMP | 0.9122±0.0679 | 0.9712±0.0327 | 4.5584±1.5822 | 0.1237±0.0539 | 0.2200 | 0.3300 | 0.0583 | 0.8491 | 0.2750 | 0.0136 |
| 13 | IEMG+RMS+WL | 0.8964±0.0760 | 0.9695±0.1126 | 4.8551±1.6050 | 0.1086±0.0694 | 0.4900 | 0.0400 | 0.0671 | 0.8216 | 0.2650 | 0.0146 |
| 14 | MAV+WAMP | 0.9090±0.0676 | 0.9779±0.0259 | 4.6828±1.5885 | 0.1093±0.0461 | 0.3800 | 0.2800 | 0.0565 | 0.8087 | 0.3300 | 0.0151 |
| 15 | MAV+WAMP+WL | 0.9114±0.0746 | 0.9681±0.0588 | 4.5964±1.6877 | 0.1222±0.0629 | 0.2100 | 0.4100 | 0.0602 | 0.8472 | 0.3100 | 0.0158 |
| 16 | IEMG+RMS+WAMP | 0.9142±0.0825 | 0.9569±0.1100 | 4.5646±1.6152 | 0.1314±0.0735 | 0.1500 | 0.4300 | 0.0644 | 0.8773 | 0.2900 | 0.0164 |
| 17 | MAV+RMS+WL | 0.8912±0.0776 | 0.9784±0.0258 | 5.0994±1.6067 | 0.1056±0.0452 | 0.1600 | 0.4600 | 0.0652 | 0.8326 | 0.3100 | 0.0168 |
| 18 | IEMG+MAV+RMS+WAMP+WL | 0.9076±0.0711 | 0.9658±0.0750 | 4.5943±1.5325 | 0.1214±0.0598 | 0.2000 | 0.4300 | 0.0633 | 0.8441 | 0.3150 | 0.0168 |
| 19 | RMS+WAMP | 0.9025±0.0903 | 0.9738±0.0249 | 4.8316±1.8639 | 0.1167±0.0453 | 0.3200 | 0.3300 | 0.0619 | 0.8485 | 0.3250 | 0.0171 |
| 20 | IEMG+MAV+RMS+WAMP | 0.9132±0.0711 | 0.9679±0.0492 | 4.6074±1.6550 | 0.1227±0.0538 | 0.2600 | 0.4600 | 0.0595 | 0.8499 | 0.3600 | 0.0182 |
| 21 | IEMG+MAV+RMS | 0.8818±0.1033 | 0.9719±0.0569 | 5.2026±1.8106 | 0.1129±0.0596 | 0.2000 | 0.3900 | 0.0732 | 0.8679 | 0.2950 | 0.0187 |
| 22 | MAV+WL | 0.8940±0.0721 | 0.9782±0.0289 | 5.0412±1.6272 | 0.1073±0.0482 | 0.1600 | 0.5700 | 0.0639 | 0.8335 | 0.3650 | 0.0194 |
| 23 | IEMG+MAV+RMS+WL | 0.9068±0.0784 | 0.9675±0.0776 | 4.7496±1.6331 | 0.1170±0.0682 | 0.3300 | 0.4100 | 0.0629 | 0.8422 | 0.3700 | 0.0196 |
| 24 | MAV+RMS+WAMP+WL | 0.9043±0.0839 | 0.9679±0.0481 | 4.6777±1.6443 | 0.1257±0.0557 | 0.3700 | 0.4300 | 0.0639 | 0.8669 | 0.4000 | 0.0222 |
| 25 | IEMG+WAMP+WL | 0.8882±0.0945 | 0.9624±0.0780 | 4.8793±1.6191 | 0.1287±0.0642 | 0.4100 | 0.2900 | 0.0747 | 0.8957 | 0.3500 | 0.0234 |
| 26 | WAMP | 0.9066±0.0683 | 0.9697±0.0499 | 4.7485±1.5699 | 0.1239±0.0597 | 0.3500 | 0.5400 | 0.0619 | 0.8668 | 0.4450 | 0.0239 |
| 27 | RMS+WAMP+WL | 0.8879±0.1092 | 0.9682±0.0569 | 4.9781±1.8215 | 0.1219±0.0629 | 0.6100 | 0.3000 | 0.0719 | 0.8801 | 0.4550 | 0.0288 |
| 28 | IEMG+RMS | 0.8788±0.0906 | 0.9746±0.0316 | 5.2628±1.8472 | 0.1146±0.0479 | 0.3900 | 0.6800 | 0.0733 | 0.8794 | 0.5350 | 0.0345 |
| 29 | MAV+RMS | 0.8497±0.1071 | 0.9768±0.0233 | 5.6081±1.9687 | 0.1132±0.0435 | 0.3900 | 0.5000 | 0.0868 | 0.9052 | 0.4450 | 0.0349 |
| 30 | IEMG+MAV+WAMP+WL | 0.8806±0.1390 | 0.9503±0.1137 | 4.9230±1.8959 | 0.1397±0.0719 | 0.4500 | 0.4700 | 0.0846 | 0.9389 | 0.4600 | 0.0365 |
| 31 | IEMG+RMS+WAMP+WL | 0.8913±0.1109 | 0.9587±0.0725 | 4.7921±1.7054 | 0.1357±0.0656 | 0.6000 | 0.5300 | 0.0750 | 0.9129 | 0.5650 | 0.0387 |

**Table SD3**-**3.** The comparison of the sEMG feature variations calculated over BF+MG+TA+RF+GMax. Green shaded cells show the feature variations that provide strongly correlated position and moment predictions (i.e., r > 0.90). BF: biceps femoris, MG: medial gastrocnemius, TA: tibialis anterior, RF: rectus femoris, GMax: gluteus maximus, IEMG: integrated EMG, MAV: mean absolute value, WAMP: Willison amplitude, RMS: root mean square; WL: waveform length.

| **Rank** | **sEMG Feature Variations** | **Position Correlation  [r]** | **Moment Correlation  [r]** | **Position RMSE  [deg]** | **Moment RMSE  [Nm/kg]** | **Position SPM [%GC]** | **Moment SPM [%GC]** | **Miscorrelation Score** | **Error  Score** | **SPM Score** | **Overall Error Score** |
| --- | --- | --- | --- | --- | --- | --- | --- | --- | --- | --- | --- |
| 1 | IEMG+WL | 0.9161±0.0792 | 0.9739±0.0445 | 4.2673±1.6024 | 0.1080±0.0547 | 0.0200 | 0.3600 | 0.0550 | 0.5897 | 0.1900 | 0.0062 |
| 2 | IEMG | 0.8929±0.0770 | 0.9746±0.0493 | 5.0677±1.7363 | 0.1095±0.0522 | 0.1700 | 0.1600 | 0.0662 | 0.6498 | 0.1650 | 0.0071 |
| 3 | MAV+WL | 0.9010±0.0700 | 0.9747±0.0659 | 4.8690±1.4697 | 0.1098±0.0594 | 0.0500 | 0.3400 | 0.0622 | 0.6367 | 0.1950 | 0.0077 |
| 4 | IEMG+MAV+RMS | 0.8874±0.0854 | 0.9751±0.0420 | 5.0965±1.6504 | 0.1120±0.0511 | 0.3000 | 0.0600 | 0.0688 | 0.6585 | 0.1800 | 0.0081 |
| 5 | RMS+WL | 0.9010±0.0856 | 0.9760±0.0339 | 4.7561±1.5845 | 0.1094±0.0550 | 0.2400 | 0.2200 | 0.0615 | 0.6277 | 0.2300 | 0.0089 |
| 6 | WL | 0.9056±0.0672 | 0.9757±0.0350 | 4.7949±1.4537 | 0.1118±0.0523 | 0.0900 | 0.4200 | 0.0593 | 0.6369 | 0.2550 | 0.0096 |
| 7 | IEMG+MAV+RMS+WL | 0.9115±0.0598 | 0.9746±0.0462 | 4.7153±1.4601 | 0.1090±0.0546 | 0.2800 | 0.2800 | 0.0570 | 0.6238 | 0.2800 | 0.0099 |
| 8 | IEMG+RMS | 0.8945±0.0801 | 0.9749±0.0378 | 4.9641±1.6571 | 0.1117±0.0532 | 0.1000 | 0.4500 | 0.0653 | 0.6485 | 0.2750 | 0.0116 |
| 9 | MAV+RMS | 0.8721±0.0916 | 0.9773±0.0232 | 5.3511±1.8434 | 0.1107±0.0432 | 0.0800 | 0.4000 | 0.0753 | 0.6729 | 0.2400 | 0.0122 |
| 10 | IEMG+MAV+WL | 0.8908±0.0810 | 0.9715±0.0606 | 4.9610±1.6300 | 0.1110±0.0602 | 0.4500 | 0.1700 | 0.0689 | 0.6464 | 0.3100 | 0.0138 |
| 11 | MAV+WAMP | 0.8927±0.1099 | 0.9614±0.0527 | 4.6629±1.7277 | 0.1368±0.0555 | 0.2700 | 0.3200 | 0.0730 | 0.6950 | 0.2950 | 0.0150 |
| 12 | IEMG+RMS+WL | 0.9034±0.0708 | 0.9774±0.0367 | 4.7590±1.5961 | 0.1093±0.0516 | 0.2600 | 0.5600 | 0.0596 | 0.6276 | 0.4100 | 0.0153 |
| 13 | IEMG+MAV | 0.8866±0.0822 | 0.9750±0.0389 | 5.1393±1.7248 | 0.1097±0.0540 | 0.2900 | 0.4600 | 0.0692 | 0.6553 | 0.3750 | 0.0170 |
| 14 | MAV+RMS+WL | 0.9029±0.0688 | 0.9744±0.0398 | 4.8618±1.4645 | 0.1120±0.0590 | 0.3000 | 0.5900 | 0.0614 | 0.6421 | 0.4450 | 0.0175 |
| 15 | RMS+WAMP+WL | 0.8916±0.1379 | 0.9542±0.1222 | 4.6949±1.7055 | 0.1332±0.0743 | 0.2100 | 0.4900 | 0.0771 | 0.6876 | 0.3500 | 0.0186 |
| 16 | MAV+RMS+WAMP+WL | 0.8986±0.1075 | 0.9341±0.1303 | 4.6209±1.5790 | 0.1605±0.0824 | 0.3100 | 0.3500 | 0.0837 | 0.7560 | 0.3300 | 0.0209 |
| 17 | WAMP | 0.8947±0.0909 | 0.9427±0.0883 | 4.8420±1.6974 | 0.1570±0.0740 | 0.2300 | 0.4800 | 0.0813 | 0.7620 | 0.3550 | 0.0220 |
| 18 | MAV+WAMP+WL | 0.8999±0.0991 | 0.9407±0.1215 | 4.6309±1.6504 | 0.1508±0.0880 | 0.2500 | 0.5400 | 0.0797 | 0.7305 | 0.3950 | 0.0230 |
| 19 | WAMP+WL | 0.9034±0.1083 | 0.9289±0.1479 | 4.5968±1.6977 | 0.1650±0.0935 | 0.3200 | 0.4700 | 0.0838 | 0.7664 | 0.3950 | 0.0254 |
| 20 | IEMG+MAV+RMS+WAMP | 0.9013±0.1133 | 0.9132±0.1452 | 4.5240±1.7302 | 0.1855±0.0902 | 0.2200 | 0.5000 | 0.0927 | 0.8166 | 0.3600 | 0.0273 |
| 21 | IEMG+RMS+WAMP+WL | 0.8983±0.1208 | 0.9294±0.1226 | 4.4664±1.6331 | 0.1807±0.0921 | 0.3600 | 0.4600 | 0.0861 | 0.7996 | 0.4100 | 0.0282 |
| 22 | IEMG+MAV+WAMP+WL | 0.8940±0.0963 | 0.9488±0.0822 | 4.6982±1.7443 | 0.1485±0.0735 | 0.3900 | 0.6000 | 0.0786 | 0.7290 | 0.4950 | 0.0284 |
| 23 | MAV+RMS+WAMP | 0.9109±0.0971 | 0.9284±0.0971 | 4.6091±1.6524 | 0.1730±0.0784 | 0.5400 | 0.3700 | 0.0804 | 0.7889 | 0.4550 | 0.0288 |
| 24 | RMS+WAMP | 0.8870±0.1165 | 0.9438±0.1421 | 5.1122±1.7414 | 0.1468±0.0768 | 0.5000 | 0.4100 | 0.0846 | 0.7534 | 0.4550 | 0.0290 |
| 25 | IEMG+WAMP | 0.8941±0.1136 | 0.9229±0.1465 | 4.7002±1.7566 | 0.1700±0.0909 | 0.3100 | 0.5000 | 0.0915 | 0.7871 | 0.4050 | 0.0292 |
| 26 | IEMG+MAV+WAMP | 0.9001±0.0850 | 0.9251±0.1476 | 4.6841±1.7421 | 0.1776±0.0872 | 0.3000 | 0.5700 | 0.0874 | 0.8065 | 0.4350 | 0.0307 |
| 27 | IEMG+MAV+RMS+WAMP+WL | 0.8749±0.1305 | 0.9370±0.1030 | 4.8256±1.8725 | 0.1616±0.0838 | 0.4100 | 0.6200 | 0.0941 | 0.7733 | 0.5150 | 0.0375 |
| 28 | IEMG+WAMP+WL | 0.8830±0.1126 | 0.9182±0.1511 | 4.8519±1.6955 | 0.1736±0.0920 | 0.4000 | 0.6300 | 0.0994 | 0.8075 | 0.5150 | 0.0413 |
| 29 | IEMG+RMS+WAMP | 0.8983±0.0847 | 0.9170±0.1728 | 4.7242±1.6463 | 0.1693±0.0966 | 0.4600 | 0.8100 | 0.0924 | 0.7869 | 0.6350 | 0.0461 |
| 30 | MAV | 0.8014±0.0983 | 0.9760±0.0246 | 6.2347±1.8979 | 0.1138±0.0445 | 0.7400 | 0.5900 | 0.1113 | 0.7430 | 0.6650 | 0.0550 |
| 31 | RMS | 0.6911±0.1083 | 0.9764±0.0240 | 7.1449±1.9921 | 0.1129±0.0439 | 0.6000 | 0.5800 | 0.1662 | 0.8043 | 0.5900 | 0.0789 |
